# Development of Transferable Coarse-Grained Lipid Models with Optimized Structural and Elastic Membrane Properties

**DOI:** 10.1021/acs.jctc.5c00579

**Published:** 2025-09-23

**Authors:** Soumil Y. Joshi, Teshani Kumarage, Rana Ashkar, Sanket A. Deshmukh

**Affiliations:** † Department of Chemical Engineering, 1757 Virginia Tech, Blacksburg, Virginia 24061, United States; ‡ Department of Physics and Center for Soft Matter and Biological Physics, Virginia Tech, Blacksburg, Virginia 24061, United States

## Abstract

Lipid membranes play crucial roles in cellular functions and offer diverse engineering applications. Studying their properties is critical but computationally demanding through atomistic simulations. In this work, we develop coarse-grained (CG) models for phosphocholine lipids, aimed at balancing computational efficiency and predictive accuracy with chemical and temperature transferability. We introduce chargeless CG beads with 2:1 or 3:1 mapping and optimize the force fields through a combination of systematic and accelerated approaches that integrate particle swarm optimization algorithm with molecular dynamics simulations. The optimization utilizes structural and elastic membrane properties obtained experimentally through X-ray and neutron scattering studies including lipid packing density, membrane thickness, and bending modulus. Validation against atomistic simulations shows that our CG models accurately reproduce the structural features of lipids including bond and angle distributions, radial distribution functions, and key bilayer properties across various system sizes and simulation time steps. A unique feature of our CG models is the bead transferability across lipids of different chain structures as well as polymeric macromolecules with similar atomic grouping. This capability facilitates future studies of more complex systems including lipid mixtures, hybrid lipid–polymer membranes, and lipid–glycomaterial complexes—thus offering an efficient platform for predicting structural and functional dynamics while mitigating the computational challenges of atomistic simulations.

## Graphical abstract

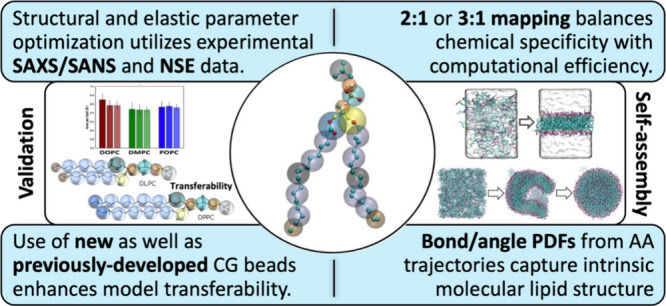

## 1 Introduction

Lipids are key components of cellular membranes serving various biological functions such as energy storage,^1,2^ signaling cascades,^3,4^ pathogen interaction immune responses,^5,6^ and more.^7–9^ Inspired by the superb multifunctionality of cellular membranes and the diversity of lipid structures, lipids have emerged as a configurable design element in many recent biotechnologies including artificial cells, gene delivery, and biosensing assays.^10–13^ These applications strongly leverage the amphiphilic nature of lipid molecules, with hydrophilic head groups and hydrophobic tails consisting of saturated or unsaturated hydrocarbon chains, which facilitates their organization into self-assembled structures, e.g., membranes or vesicles, in aqueous environments.^14,15^ Furthermore, variations in the lipid headgroup, hydrocarbon chain length, or degree of chain unsaturation offer a remarkable toolkit for structural, dynamical, and functional tunability.^16–18^ Synergistically, computational modeling of lipids and their assemblies has provided significant mechanistic insights into cellular processes and continues to guide the design of synthetic membranes with targeted performance. Indeed, the convergence of computational modeling and experimental studies in recent years have significantly advanced our understanding of lipid self-assemblies into membranes, revealing crucial details such as molecular arrangements, elastic responses, and electrostatic properties.^19–22^ What is more, this synergy has been central to the development of more accurate, predictive, computational models that can effectively inform molecular designs of lipid membranes and related biomimetic materials with tunable characteristics.^23,24^

Computational techniques like molecular dynamics (MD) simulations are often used to study lipid membranes at the molecular level,^20,25,26^ and are very well reviewed in the literature.^27,28^ All-atom (AA) models for lipids defined using CHARMM,^29–31^ and AMBER force fields (FFs)^32,33^ explicitly capture every atom in the system, offering the most complete molecular representation.^34–36^ The GROMOS FF,^37^ on the other hand, employs a united atom (UA) approach representing each of nonpolar CH, CH_2_ and CH_3_ groups of hydrocarbons as single particles allowing it to reach a 3-fold speedup compared to AA simulations. Overall, AA and UA MD simulations have been successfully used to reproduce and predict various membrane properties^25^ including the average area per lipid,^38^ membrane thickness,^39^ order parameter,^40^ bending rigidity,^20,41^ and polarization effects.^42,43^ However, these simulations require substantial computational resources, and generally use smaller time steps,^44–47^ limiting their use in studying slower processes like membrane fusion and molecular permeation at biologically relevant time scales.^48^

On the other hand, the use of coarse-grained (CG) models, where multiple atoms are combined into larger CG beads, circumvents these limitations by lowering the system sizes and allowing the use of longer time steps due to reduced degrees of freedom.^49–51^ Thus, CG MD simulations are widely considered to simulate large and complex lipid systems over longer time scales.^52–56^ The caveat is that coarse-graining requires the development of force fields that can reliably predict lipid membrane properties, making it a crucial first step. In the past, CG lipid models have been developed using energy-based, force-matching, or structure-based approaches.^48,57–59^ The MARTINI lipid CG model is a notable example, developed using the partitioning free energies between polar and apolar phases of a large number of chemical compounds.^60^ Due to its computational efficiency, and ability to capture essential biomolecular interactions, the MARTINI FF has been utilized to study thermodynamic, dynamic, and structural properties of lipid rafts,^61,62^ lipid membranes,^63–65^ and lipoprotein particles.^66,67^ The multiscale coarse-graining (MS-CG) approach developed by Izvekov and Voth systematically utilizes forces obtained from AA simulations to formulate a generalized parametrization.^68–70^ In the case of structure-based CG models of lipids, they have been parametrized to reproduce several structural properties including membrane thickness, order parameters, electron densities, and radial distribution functions (RDFs) obtained from experiments and AA MD simulations. Similarly, other lipid CG models have been developed with specific features, such as implicit solvent representation,^71–75^ soft-repulsive core representation (dissipative particle dynamics models),^76–78^ machine learning (ML)-based,^79,80^ and mixed resolution.^81,82^ Although models developed using these approaches have been used widely, most of them exhibit limited transferability and/or compatibility across FFs, which is nonideal for studies of complex or hybrid assemblies requiring simultaneous modeling of lipids and other macromolecules.^55,83^ They employ coarse mapping schemes, and a lack of systematic comparisons with AA-mapped trajectories leads to a significant loss of critical physical and chemical details.^84^ Furthermore, a few models use complex FF equations that are not readily implementable in commonly used simulation softwares.

Here, we develop new CG models for phosphocholine (PC) lipids optimized using experimental observations of both structural and elastic membrane properties. We specifically note that we optimize the model parameters to simultaneously reproduce structure and mechanics, providing a unified framework to better capture the correlation between the structural features and elastic responses. This has direct implications in various membrane processes including dynamic membrane responses to external stimuli, membrane interactions with proteins or additive molecules, or designs of stable liposomal carriers.^85^ Our models have the following features: (*i*) They reproduce key membrane structural properties (i.e., area per lipid, *A*_L_, and phosphate-to-phosphate thickness, *D*_PP_) as well as elastic properties (i.e., bending modulus, κ) by optimizing against findings from small-angle X-ray/neutron scattering (SAXS/SANS) experiments and neutron spin echo (NSE) spectroscopy studies. This combination of structural and elastic properties enables us to model membranes with enhanced physical realism, capturing both their equilibrium structure and mechanical response under varying conditions. (*ii*) They consist of 2:1 or 3:1 mapped beads, to optimally capture the inherent chemistry of these molecules while simultaneously making the models computationally efficient. (*iii*) They utilize a combination of new and previously developed CG beads, making our models chemically transferable.^49,84,86–91^ (*iv*) They are specifically optimized to incorporate interactions with an explicit 1-site water model, to potentially capture solvent effects in various processes. (*v*) They employ commonly implemented harmonic potentials and 12–6 nonbonded Lennard-Jones interactions, making them compatible with commonly used MD simulations packages such as Nanoscale Molecular Dynamics (NAMD),^92^ and Groningen Machine for Chemical Simulation (GROMACS).^93^ (*vi*) They leverage mapping of bond angle distributions and RDFs, obtained from AA-mapped trajectories, to better capture the conformational properties of lipid chains. This comparison is crucial for accurately capturing the intrinsic structural behavior of individual lipids,^84^ which complements the membrane-level structural properties like area per lipid (*A*_L_) and phosphate-to-phosphate thickness (*D*_PP_).

In Section 2, we describe the methods used for model development and the experimental procedure for data collection. Section 3 evaluates the performance of our optimized CG models by comparing them with atomistic simulations and experimental data. We further validate our models through systematic CG MD simulations, assessing their behavior across different timesteps and system sizes while also examining the impact of parameter perturbations on key properties. Additionally, we demonstrate the ability of our CG models to spontaneously self-assemble into biologically relevant structures, such as lipid bilayers and vesicles. We also evaluate the chemical and temperature transferability of our models using lipid molecules not included in the development process. Here we note that the present model does not include explicit electrostatics, yet it successfully reproduces the self-assembly and structural properties of zwitterionic PC lipids, where hydrophobic and steric interactions are primarily responsible for bilayer formation and physical observables. This design choice is consistent with prior experimental and modeling studies on such systems.^53,55,94–96^ Future extensions of the model will incorporate electrostatics explicitly, allowing application to charged lipids, mixed compositions, and membrane-ion or membrane-protein systems. Finally, in Section 4, we present our conclusions, discuss limitations, and propose strategies for further improvement.

## 2 Methodology

### 2.1 Model Development Overview

In this work, we parametrize CG beads for three types of PC lipids, namely 1,2-dioleoyl-*sn*-glycero-3-phosphocholine (DOPC), 1-palmitoyl-2-oleoyl-glycero-3-phosphocholine (POPC), and 1,2-dimyristoyl-*sn*-glycero-3-phosphocholine (DMPC). The selected lipids encompass a range of chain lengths and degrees of unsaturation, leading to distinct membrane properties that could be measured both experimentally and through simulations. The selection of these specific lipids is further utilized for the development of temperature-transferable models. Specifically, due to the different transition temperatures of the three lipids, our experimental data were collected at 25 °C (298 K) for DOPC and POPC lipid membranes whereas DMPC membranes were measured at 44 °C (317 K), i.e., 20 °C above the melting transition. Consequently, the FF parameters for CG models are optimized by performing CG MD simulations at both 298 and 317 K. Using the NAMD package,^92^ in combination with Particle Swarm Optimization (PSO), we utilize a hybrid parametrization approach, integrating structural characteristics from AA MD simulations with experimentally measured properties.

#### 2.1.1 Mapping Scheme

Here, we use 2:1 or 3:1 mapped beads (4:1 if absolutely necessary as in the case of 2/10 beads) as presented in Figure 1A. The mapping scheme is chosen to reasonably capture the chemical identities of different subgroups of the lipid molecule (hydrophilic heads, hydrophobic tails, etc.) using similar CG representations. The CG lipid models are developed by making use of existing transferable beads, previously developed in our group for hydrocarbon (beads C2M, C2E, C3M and C3E) and amino acid (bead CCO) models, as well as new CG beads.^87–90^ As shown in Figure 1A, the new CG beads include a PO2 bead that encompasses a phosphorus and two oxygens (3:1 mapping scheme), a CHO bead for the choline headgroup (4:1 mapping scheme), and a COH bead encompasses two oxygens and a carbon (3:1 mapping scheme), and an MTF bead with two carbons and two oxygens (4:1 mapping scheme) in PC lipids. Additionally, a D3M bead is defined to represent unsaturated segments of the hydrocarbon chains with the goal of capturing the effect of unsaturation on bonded and nonbonded interactions. A NAMD topology file for generating systems using the current mapping scheme is presented at the end of the Supporting Information (SI).

**Figure 1:**
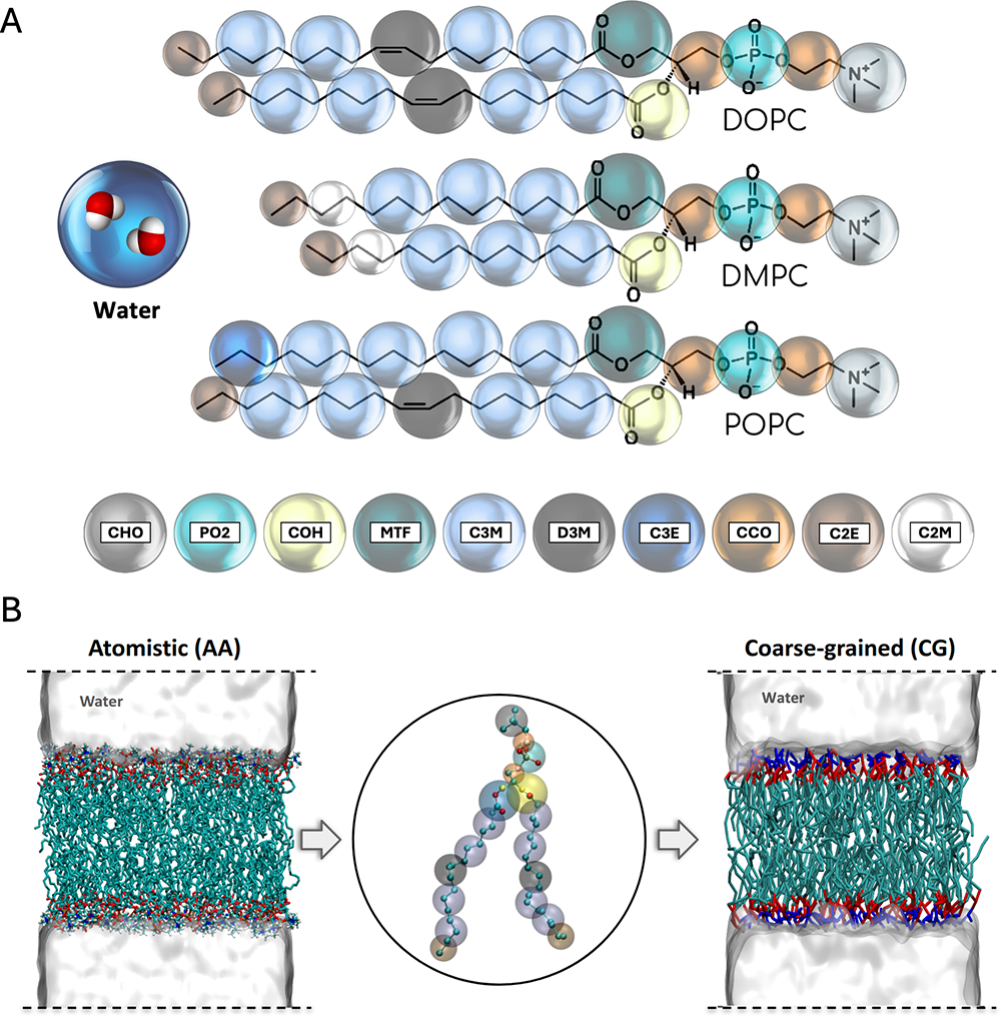
**A)** Mapping schemes for the lipid molecules (DOPC, POPC, DMPC) as well as water, implemented in this work. Bead types are presented at the bottom of the figure, and beads with the same color correspond to the same bead type or chemically transferable beads. For lipids, hydrogen atoms are not shown for most carbons for the sake of clarity, but they are included in the bead mapping. Water CG beads contain two molecules per bead. **B)** Representative snapshots of initial configurations used for running atomistic and CG MD simulations in this study, along with the CG mapped structure of DOPC as an example in the inset. Initial configuration snapshots are colored as follows: headgroups are colored blue, regions representing the glycerol backbone are colored red, and hydrocarbon chains are colored cyan.

#### 2.1.2 Force Field Equation and Parameterization Strategy

The following FF defines the bonded and nonbonded interactions between the different bead types in our systems:

1 $$E_{pot}=K_{b}{\left(b-b_{0}\right)}^{2}+K_{\theta }{\left(\theta -\theta_{0}\right)}^{2}+4\varepsilon \left[{\left(\frac{\sigma }{r_{ij}}\right)}^{12}-{\left(\frac{\sigma }{r_{ij}}\right)}^{6}\right]$$

The first two terms on the right-hand side of eq 1 represent bonded parameters, namely bond and angle potentials. Dihedrals and impropers are excluded from the models in order to enable the use of longer time steps by reducing the degrees of freedom in the simulated molecules. The harmonic potential form captures bonded parameters where *K*_*b*_ and *K*_*θ*_ represent the bond and angle force constants, respectively. As part of our hybrid approach, equilibrium bond (*b*_0_) and angle (*θ*_0_) values, as well as their respective force constants are obtained by comparison with AA mapped bond and angle distributions.

The 12–6 functional form of Lennard–Jones (LJ) potentials defines nonbonded interactions between CG beads (eq 1) where *σ* represents the finite distance resulting in a zero interbead potential, *ε* represents the strength between interbead interactions,^87^ and *r*_*ij*_ is the distance between two CG beads *i* and *j*. All CG beads are set to have zero charge. The Lorentz–Berthelot (LB) mixing rule dictates cross-interactions between CG beads if interactions are not explicitly optimized.^97^ Beads separated by at least two bonds are allowed to interact through nonbonded interactions. This ensures consistency between the developed lipid models and our previous models of solvents, polymers, and biomolecules thus maintaining chemical transferability for eventual integration with other systems.^84,86–91,98,99^ Nonbonded self-interactions of the new CG beads (PO2, CHO, COH, MTF) and their interactions with water are optimized using experimentally obtained bilayer properties. Specifically, we use structural parameters obtained from SAXS/SANS studies as well as elastic parameters obtained from NSE spectroscopy studies.

### 2.2 Properties of Interest

The current work aims to develop CG models that can reproduce not only regularly modeled structural properties of lipid membranes but also their elastic properties that are often overlooked in computational studies. Specifically, we report the efficacy of our models in capturing experimental bending moduli (κ) of different lipid bilayers alongside their structural properties.^100–105^

#### 2.2.1 Experimental Measurements

Structural membrane parameters obtained from high-resolution small-angle X-ray and neutron scattering (SAXS/SANS) measurements are described in earlier studies.^100–105^ Such measurements yield the phosphate-to-phosphate thickness (*D*_PP_), the total membrane thickness (*D*_B_), and the average area per lipid (*A*_L_) from unilamellar vesicles (ULVs) of lipid membranes in protiated/deuterated buffer for SAXS/SANS studies, respectively. Details about the experimental conditions can be found in the SI. The collected data from SAXS and SANS are simultaneously analyzed using a modified scattering density profile (SDP) model, the details of which are elaborated in prior studies by Heberle et al.,^102^ Chakraborty et al.,^20^ and Kumarage et al.^106^ The theoretical framework underlying this analysis can be found in the referenced studies in Section S1 in the SI. Membrane bending moduli (κ) are measured by neutron spin echo (NSE) spectroscopy.^20,107,108^ These experiments, performed on ULVs of lipid membranes in a deuterated buffer, allow for probing membrane bending fluctuations over length scales of ∼70–200 Å and time scales of ∼1–100 ns.^20,107,108^

#### 2.2.2 Computational Calculations from CG MD Simulations

The structural parameters *A*_L_ and *D*_PP_ are obtained using an in-house Python script from multiple frames of MD trajectories. *A*_L_ is calculated by dividing the cross-sectional area of the simulated bilayer by the number of lipids in each leaflet.^109^ Similarly, *D*_PP_ is calculated from the perpendicular distance between PO2 beads (Figure 1) in both leaflets.^109^ κ is calculated through the real-space fluctuation (RSF) analysis method introduced by Doktorova et al.^41,110^ This method estimates κ from the distribution of local splay angles, defined as the deviation between a lipid tail vector and the interpolated local bilayer normal. The probability distribution *P*(*S*) of splay angles follows a Boltzmann form:

2 $$P\left(S\right)=C\times \exp\left(-\frac{\kappa S^{2}A_{L}}{2k_{B}T}\right)$$

where *S* is the splay angle, *A*_L_ is the area per lipid, and *C* is a normalization constant. Rearranging this, κ can be extracted by fitting the potential of mean force (PMF) as^41^

3 $$\frac{-2k_{B}T}{A_{L}}\times \ln\left(P\left(S\right)\right)=\kappa S^{2}+C^{\prime }$$

To ensure robust fitting, the RSF method repeats the quadratic fit across five different windows centered around the *P*(*S*) mean (μ), with ranges of ±1σ to ±2σ. κ is reported from the ±1σ fit, and associated uncertainty is the standard deviation of the five κ values obtained.^111^ Unlike Fourier-based undulation analyses that require large system sizes to capture long-wavelength modes, the localized nature of the RSF method enables extraction of bending moduli even for small or multicomponent bilayer systems.^41,110^ Because the method uses splay angles from all lipids across the sampled trajectory, it provides comprehensive sampling even in modestly sized systems as has been successfully validated for bilayers with sizes comparable to those used in our CG simulations.^41,110–113^ The analysis utilizes the Openstructure software^114^ and Python modules developed by Johner et al.^111^

### 2.3 Parameter Optimization

Considering the large numbers of parameters to be optimized, bonded and nonbonded parameters are optimized in two different stages. Generally in CG models, structural properties such as bond and angle distributions can be considered independently of nonbonded parameters, which primarily determine the bulk properties of interest in our work.^49^ Consequently, our approach starts by optimizing bonded parameters, with the focus on accurately capturing the structural characteristics obtained from mapped AA trajectories. Next, the nonbonded parameters are tuned to satisfactorily reproduce the properties of interest using these optimized bonded parameters. Once these properties are obtained both bonded and nonbonded parameters get further validated by comparing with mapped trajectories and experimental data.

#### 2.3.1 Bonded Parameters

Mapped bond and angle distributions, obtained from atomistic simulations of DOPC, POPC, and DMPC are used as targets while optimizing force constants, and the equilibrium bond and angle values in the CG model. Atomistic trajectories consisting of 1000 frames, with a simulation time span of 5 ns are mapped to their corresponding CG representations by calculating the centers of mass of all atoms (including hydrogens) encompassed within each CG bead. The mapped trajectories are visualized using the Visual Molecular Dynamics (VMD) package,^115^ while bond and angle distributions are calculated using an in-house TCL script. For existing beads, their bonded and nonbonded parameters are used without modification. For newly introduced beads, bonded parameters are initially set to the mean of the bonded distributions obtained from mapped AA trajectories, with force constants chosen to reproduce these distributions. All bond and angle distributions are recalculated after optimizing the nonbonded parameters to ensure the continued validity of the bonded interaction parameters. Any further adjustments are validated by comparing the results with mapped AA trajectories and experimental data. Specifically, 12 bond and 16 angle parameters are optimized during this step, as shown in Table S1 in the SI.

#### 2.3.2 Nonbonded Parameters

Nonbonded interactions are represented by 12–6 LJ potential with ε signifying the strengths of nonbonded interactions and σ signifying the equilibrium interbead distances between interacting CG beads. Here, we develop both *ε* and *σ* parameters for five self-interaction, and four cross-interaction potentials shown in Table S2 in the SI. AA-mapped RDFs are plotted for beads CHO, PO2, COH, MTF, and D3M, using the mapped trajectories of DOPC, DMPC, and POPC generated while optimizing bonded parameters. RDFs for the three lipids are averaged, and first peak positions are used to formulate narrow ranges for the optimization of *r*_min_ for self-interaction parameters. Optimized *r*_min_ values are used to obtain respective σ values through the following $r_{\min}=2^{1/6}\sigma$ .

Initial ranges for optimizing the various self-interaction *ε* values are estimated by considering the polarities of atoms represented in the CG beads as well as through intuitive comparisons with previously developed models.^49^ The maximum lower bound for these *ε* values is typically −1.1 kcal/mol, in the case of the ‘strongest’ interactions, while the upper bound is typically around −0.5 kcal/mol, for the weakest interactions. In case of cross-interactions with water, ranges for *ε* are obtained by calculating the LB mixing rule values for every new CG bead at the upper and lower bounds of their respective self-interaction parameter ranges. For cross-interaction σ values, considering the narrow ranges of self-interaction *σ* parameters, wider ranges are used than the bounds obtained from the LB combining rule. This allows us to carry out an extensive search of the parameter space for tuning interactions between the new CG beads and water, while also partly relying on LB combining rules. We also allow larger ranges for cross-interaction *σ* values, to balance out model properties while being rigid with tuning the self-interaction parameters. Initial ranges used for nonbonded parameter optimization are shown in Table S2 in the SI.

PSO runs are initialized with 5 particles (5 sets of FF parameters), wherein simulations for DOPC, POPC, and DMPC are carried out simultaneously as they are composed of common beads, enabling us to develop chemically transferable CG models. Simulations are performed for 25 ns, of which the final 5 ns are considered the production run and used for calculating *A*_L_, *D*_PP_, and κ for all three lipid systems. These are compared to the target values obtained from experiments and overall error is calculated for further refinement. Weights of 1.5, 1.2, and 1.0 are assigned to *A*_L_, *D*_PP_, and κ, respectively, to prioritize the error reduction in structural properties, with *A*_L_ being weighted the highest. Extensive details regarding the PSO method and its impact on parameter optimization can be found in Section S2 of the SI and in our previous works.^86,87^ All newly optimized nonbonded parameters, including 5 self- and 4 cross-interaction parameters, are presented in Table S2. Additionally, a parameter file for the developed CG lipid FF which can be used directly with the NAMD simulation package is presented towards the end of the SI and can also be downloaded from here: https://github.com/Deshmukh-Group/CG-Lipid-model.

### 2.4 MD Simulation Protocol

A number of AA and CG MD simulations are performed using multiple lipid systems, for generating mapped trajectories, optimizing interaction parameters through PSO, running tests to validate model performance, or studying model robustness and capabilities. Unless otherwise mentioned, the general protocols implemented for running AA and CG MD simulations are described below.

#### 2.4.1 AA Simulation Protocol

Atomistic DOPC, POPC, and DMPC bilayer systems with 128 lipids along with ∼45 waters per lipid (∼6000 TIP3P water molecules), are generated using the CHARMM-GUI online package, with well-equilibrated initial structures built at the experimental *A*_L_.^116,117^ System sizes of 128 lipids were chosen to balance statistical accuracy and computational efficiency. Based on best practices outlined for simulating lipid membranes, a simulation run of at least 10 ns is essential for preassembled bilayers of this size to equilibrate.^118^ Thus, AA simulations are carried out for 25 ns, with the last 5 ns considered as the production run for the evaluation of mapped molecular characteristics. A representative snapshot for the initial configurations of the AA lipid systems is shown in Figure 1B. The bilayer systems with water measure ∼65 Å × 65 Å × 90 Å in the *x*, *y*, and *z* directions. Simulations are performed at appropriate temperatures for consistency with our experimental data. Three-dimensional periodic boundary conditions (PBC) are implemented. The NAMD 2.13 simulation package^92^ is used for running MD simulations where the CHARMM36 atomistic FF defines the interaction parameters between different atoms in the systems.^29^ Following a 1000 step minimization, the isothermal–isobaric (NPT) ensemble is used to carry out MD simulations, where temperature and pressure control is achieved using the Langevin thermostat and barostat, respectively.^119,120^ The cutoff for nonbonded interactions is set to 12 Å with a scaled 1–4 exclusion parameter, and the Particle Mesh Ewald (PME) algorithm is used to define electrostatic interactions.^121^ A 1 fs time step is utilized for the simulations.

#### 2.4.2 CG Simulation Protocol

To conduct CG MD simulations, the equilibrated bilayers from the three AA MD simulation trajectories are coarse grained using the residue-based coarse graining (RBCG) methodology incorporated in VMD, which groups atoms of particular residues into virtual beads based on their centers of mass.^122^ These bilayers are solvated using 1-site CG water beads (∼5500 beads) to create a system measuring ∼64 Å × 64 Å × 120 Å in the *x*, *y*, and *z* directions.^86^ The system dimension is extended in the *z*-direction as compared to AA simulations to incorporate larger layers of bulk water due to the reduced system sizes in CG systems. Figure 1B displays a snapshot of the initial configurations for the CG systems. CG MD simulations are performed at temperatures consistent with our experimental data and AA runs, using the NAMD 2.13 package.^92^ PBC is implemented in all three directions. The cutoff for nonbonded interactions is similarly set to 12 Å, but with a 1–2 exclusion for the CG systems. A time step of 10 fs is used to perform CG MD simulations unless otherwise specified. CG MD simulations are performed for a minimum of 25 ns,^118^ following a 1000 step minimization conducted using the default conjugate gradient and line search algorithm implemented in NAMD.^92^ The NPT ensemble is used with the Langevin thermostat and barostat for temperature and pressure control.^119,120^ Production run trajectories are utilized to analyze membrane properties—*A*_L_, *D*_PP_ and κ.

### 2.5 Model Robustness and Testing

After obtaining the optimized nonbonded parameters via the PSO algorithm, which minimizes the weighted error between simulated and experimental *A*_L_, *D*_PP_, and κ values, we performed longer CG MD simulations to validate these parameters. While the PSO optimization uses short 25 ns simulations (last 5 ns production) to quickly assess performance, the validation step involves 1 μs simulations on the same systems using the optimized parameters. Membrane properties are further calculated using the following sampling strategies: (i) 10 ns segments are sampled over 40–50 ns, 90–100 ns, 140–150 ns, and 190–200 ns intervals from longer simulation trajectories to calculate membrane properties as block averages, (ii) the final 800 ns are entirely considered for calculating membrane properties, and the mean of all block averages is calculated for all systems.

The performance of the developed lipid models is evaluated against simulation variables such as system sizes and simulation time steps. Next, to investigate the sensitivity of our calculated membrane properties, we use a Sobol sequence to perturb the optimized parameters within 5% bounds, and 5000 new sets of parameters are generated to perform CG MD simulations.^123^ The advantage of the Sobol sequence is that it performs quasi-random sampling to provide uniformly distributed parameter sets as compared to random sampling methods.^124^ Additionally, the optimized FF is tested for evaluating our models’ capabilities to (*i*) yield realistic self-assembled structures such as bilayers and vesicles from randomly mixed initial configurations, (*ii*) model other lipids not used in the model development process to assess the chemical transferability of our model, and (*iii*) reproduce temperature-dependent behavior of lipids to evaluate the temperature transferability of the model. While this section aims to only introduce the various tests performed in this study, additional details are provided alongside their respective results in the forthcoming sections.

## 3 Results and Discussions

### 3.1 Structural and Elastic Properties of Simulated Lipid Membranes

We developed optimized FF parameters using the PSO to replicate experimentally obtained *A*_L_, *D*_PP_, and κ values for DOPC, POPC, and DMPC bilayers. Properties obtained from PSO, and corresponding errors are presented in Table 1. The errors range from 0.3% to 15%, which are deemed acceptable for transferable CG models.

**Table 1:** Structural and Elastic Properties of DOPC, POPC, and DMPC Bilayers Simulated Using Optimized CG Interaction Parameters^a^

	DOPC (298 K)			POPC (298 K)			DMPC (317 K)		
128 lipid system	*A*_L_ (Å^2^)	*D*_PP_ (Å)	κ (*k*_B_ *T*)	*A*_L_ (Å^2^)	*D*_PP_ (Å)	κ (*k*_B_ *T*)	*A*_L_ (Å^2^)	*D*_PP_ (Å)	κ (*k*_B_ *T*)
**Experimental target values**	66.3 ± 1.6	34.0 ± 1.3	20.4 ± 2.5	62.7 ± 0.5	35.5 ± 0.4	23.4 ± 3.8	63.1 ± 1.1	31.6 ± 0.8	28.1 ± 4.4
**Results obtained during 25 ns parameter development cycles using PSO**									
20–25 ns	66.1	39.1	21.8	65.6	38.2	21.2	63.4	33.3	27.6
**Error (Exp vs PSO)**	0.3%	15%	6.8%	4.6%	7.6%	9.4%	0.5%	5.4%	1.8%
**Results obtained during 1000 ns validation run**									
40–50 ns	66.8 ± 0.7	39.9 ± 0.5	23.6 ± 1.1	65.3 ± 0.7	39.2 ± 0.5	24.8 ± 0.6	65.2 ± 0.9	34.3 ± 0.5	25.7 ± 0.7
90–100 ns	66.8 ± 0.5	39.8 ± 0.5	22.0 ± 0.7	65.4 ± 0.9	39.1 ± 0.4	28.1 ± 0.6	64.5 ± 0.8	34.5 ± 0.4	29.0 ± 0.8
140–150 ns	67.2 ± 0.7	39.7 ± 0.4	22.1 ± 0.5	65.1 ± 0.5	39.3 ± 0.5	26.1 ± 0.8	64.7 ± 0.8	34.5 ± 0.4	26.6 ± 0.7
190–200 ns	67.0 ± 0.8	39.7 ± 0.4	20.3 ± 0.5	65.3 ± 0.6	39.2 ± 0.3	25.3 ± 0.5	64.7 ± 0.6	34.4 ± 0.3	27.3 ± 0.7
200–1000 ns	66.2 ± 0.6	39.4 ± 0.4	21.2 ± 0.7	65.4 ± 0.5	39.3 ± 0.3	25.7 ± 0.6	64.5 ± 0.7	34.1 ± 0.3	26.3 ± 0.4
**Mean**	66.8 ± 1.5	39.7 ± 1.0	21.8 ± 1.6	65.3 ± 1.4	39.2 ± 0.9	26.2 ± 1.4	64.7 ± 1.7	34.4 ± 0.9	27.0 ± 1.5
**Error (Exp vs Mean)**	0.8%	17%	6.8%	4.1%	10%	12%	2.5%	8.9%	3.9%

a Experimental target values are shown for reference. Results are reported using distinct sampling strategies: (i) 5 ns production run from 25 ns simulations performed during the PSO, (ii) block averages calculated over four 10 ns segments from the initial 200 ns and calculated across final 800 ns from our validation run, with “Mean” values representing the averages and standard deviations across all validation blocks. All reported values remain within acceptable error margins and do not exhibit drift over time.

#### 3.1.1 Validating Interaction Parameters

Longer simulations performed on the 128 lipid bilayer systems for DOPC, POPC, and DMPC bilayers exhibit good stability and reproducibility of key structural and elastic properties across multiple checkpoints, including 40–50 ns, 90–100 ns, 140–150 ns, and 190–200 ns intervals, as presented in Table 1. The minor variations observed across time blocks reflect natural fluctuations in membrane properties during MD simulations, which can arise from differences in local lipid packing or subtle bilayer rearrangements. Importantly, these variations remain within acceptable limits and do not indicate systematic drift or model degradation. Furthermore, properties extracted for extended 800 ns trajectories show similarly consistent agreement with our experimental values, reinforcing the robustness and reliability of the optimized CG model over longer time scales. Similarly, the mean values calculated for the three lipids, averaged over our different samples, display good agreement with our experimental target values as well as those obtained during PSO integrated CG MD simulations (Table 1). For comparison, we show in Table 2 the agreement of our experimental and simulated results with previous studies. Interestingly, we find that most of the AA and CG MD simulations, including ours, overestimate the *D*_PP_ values as compared to experimental results, which has been previously attributed to tighter packing in simulated bilayers.^125^ However, in our models, this overestimation does not appear to result from tighter lateral packing, as no significant decrease in *A*_L_ is observed. Hence, to further investigate we look at bond and angle probability density functions between mapped AA and CG trajectories in section below.

**Table 2:** Calculated *A*_L_, *D*_PP_, and κ Values from This Study Compared with Published Literature^a^

**DOPC**								
***A***_**L**_ (Å^2^)			***D***_**PP**_ (Å)			**κ** (*k*_B_ *T*)		
**Value**	**Temp**	**Method**	**Value**	**Temp**	**Method**	**Value**	**Temp**	**Method**
66.25 ± 1.60	298.0	***This work (Exp)***	34.00 ± 1.29	298.0	***This work (Exp)***	20.4 ± 2.5	298.0	***This work (Exp)***
67.6 ± 0.4	298.0	SAXS/SANS^20^	35.20 ± 0.06	298.0	SAXS/SANS^20^	19.05 ± 0.65	298.0	NSE^20^
68.2 ± 0.9	298.0	AA MD (CHARMM36)^20^	38.5 ± 0.04	298.0	AA MD (CHARMM36)^20^	18.3 ± 0.3	298.0	AA MD (CHARMM36)^20^
60.0 ± 0.5	303.0	AA MD (GROMOS)^37^	30.5	303.0	AA MD (GROMOS)^37^	27.8 ± 0.7	298.0	AA MD (CHARMM36)^126^
69.0 ± 0.3	303.0	AA MD (Lipid14)^32^	37.0 ± 0.2	303.0	AA MD (Lipid14)^32^	37.4 ± 0.97	300.0	CG MD (MARTINI)^127^
69.54 ± 0.15	300.0	CG MD^128^	38.20 ± 0.08	300.0	CG MD^128^	30.0 ± 1.0	300.0	CG MD^128^
66.95 ± 1.4	298.0	***This work (CG MD)***	39.78 ± 0.9	298.0	***This work (CG MD)***	22.0 ± 1.5	298.0	***This work (CG MD)***

**POPC**								
***A***_**L**_ (Å^2^)			***D***_**PP**_ (Å)			**κ** (*k*_B_ *T*)		
**Value**	**Temp**	**Method**	**Value**	**Temp**	**Method**	**Value**	**Temp**	**Method**
62.66 ± 0.54	298.0	***This work (Exp)***	35.48 ± 0.44	298.0	***This work (Exp)***	23.4 ± 3.8	298.0	***This work (Exp)***
64.4 ± 1.3	303.0	SAXS/SANS^129^	36.5	303.0	SAXS/SANS^129^	19.0 ± 2.0	295.0	NSE^130^
63.4 ± 0.4	300.0	AA MD (CHARMM36)^131^	39.3 ± 0.2	300.0	AA MD (CHARMM36)^131^	31.7 ± 1.0	303.0	AA MD (CHARMM36)^25^
60.5 ± 0.1	300.0	AA MD (GROMOS)^131^	39.0 ± 0.1	300.0	AA MD (GROMOS)^131^	24.3 ± 0.5	298.0	AA MD (CHARMM36)^41^
64.6 ± 0.1	300.0	AA MD (Lipid14)^131^	38.3 ± 0.2	300.0	AA MD (Lipid14)^131^	30.3 ± 1.1	298.0	CG MD (MARTINI)^132^
64.0 ± 0.1	300.0	CG MD (Dry MARTINI)^71^	42.1 ± 0.5	300.0	CG MD (Dry MARTINI)^71^	-	-	-
65.28 ± 1.4	298.0	***This work (CG MD)***	39.2 ± 0.9	298.0	***This work (CG MD)***	26.1 ± 1.3	298	***This work (CG MD)***

**DMPC**								
***A***_**L**_ (Å^2^)			***D***_**PP**_ (Å)			**κ** (*k*_B_ *T*)		
**Value**	**Temp**	**Method**	**Value**	**Temp**	**Method**	**Value**	**Temp**	**Method**
63.14 ± 1.09	317.0	***This work (Exp)***	31.58 ± 0.84	317.0	***This work (Exp)***	28.1 ± 4.4	317.0	***This work (Exp)***
63.3 ± 1.3	323.0	SAXS/SANS^129^	32.2 ± 0.6	323.0	SAXS/SANS^129^	30.1 ± 2.2	313.0	^31^P NMR^133^
61.2 ± 0.2	320.0	AA MD (CHARMM36)^131^	35.8 ± 0.1	320.0	AA MD (CHARMM36)^131^	30.5	313.0	NSE^108^
60.2 ± 0.2	320.0	AA MD (GROMOS)^131^	34.5 ± 0.1	320.0	AA MD (GROMOS)^131^	34.2 ± 2.4	310.0	CG MD (Dry MARTINI)^71^
63.5 ± 0.2	320.0	AA MD (Lipid14)^131^	34.7 ± 0.1	320.0	AA MD (Lipid14)^131^	10.4 ± 0.3	300.0	CG MD^128^
61.59 ± 0.4	300.0	CG MD^128^	34.67 ± 0.03	300.0	CG MD^128^	-	-	-
64.8 ± 1.6	317.0	***This work (CG MD)***	34.43 ± 0.8	317.0	***This work (CG MD)***	27.15 ± 1.5	317.0	***This work (CG MD)***

a Also see Figures S2–S4 for better visual interpretation and discernment of trends.

### 3.2 Mapped Molecular Characteristics

Once the optimized models are tested for their structural and mechanical properties, their molecular characteristics are verified to ascertain whether the CG lipids accurately represent their atomistically mapped structures.

#### 3.2.1 Bond and Angle Probability Density Functions

Bond length and angle probability density functions (PDFs), generated from 25 ns CG simulations for DOPC, POPC, and DMPC are compared to those obtained from similarly sampled 25 ns long atomistic trajectories. Figure 2 presents representative bond and angle PDFs calculated from and averaged over all three lipid systems. Similarly, the PDFs of all bond lengths optimized in this study are presented in Figure S5 in the SI. The overlap coefficients (OCs), which represent the percentage of shared area between the CG and AA PDFs on a scale from 0 (no overlap) to 1 (perfect overlap), are presented for quantitative comparison.^134^ This metric has been previously used in other fields to evaluate model predictions and uncertainties.^135–137^ Most CG bond length PDFs (9 out of 12 distributions) showed reasonable match with their AA mapped counterparts, in terms of both the location and width of the bond distributions, as shown by OCs greater than 0.5. This was achieved by systematically tuning bond and angle parameters to best match AA mapped distributions. However, some deviations are observed for bonds closer to the lipid headgroups, such as the CCO–CHO bond (Figure 2A), where the bond PDF is shifted to the right.

**Figure 2:**
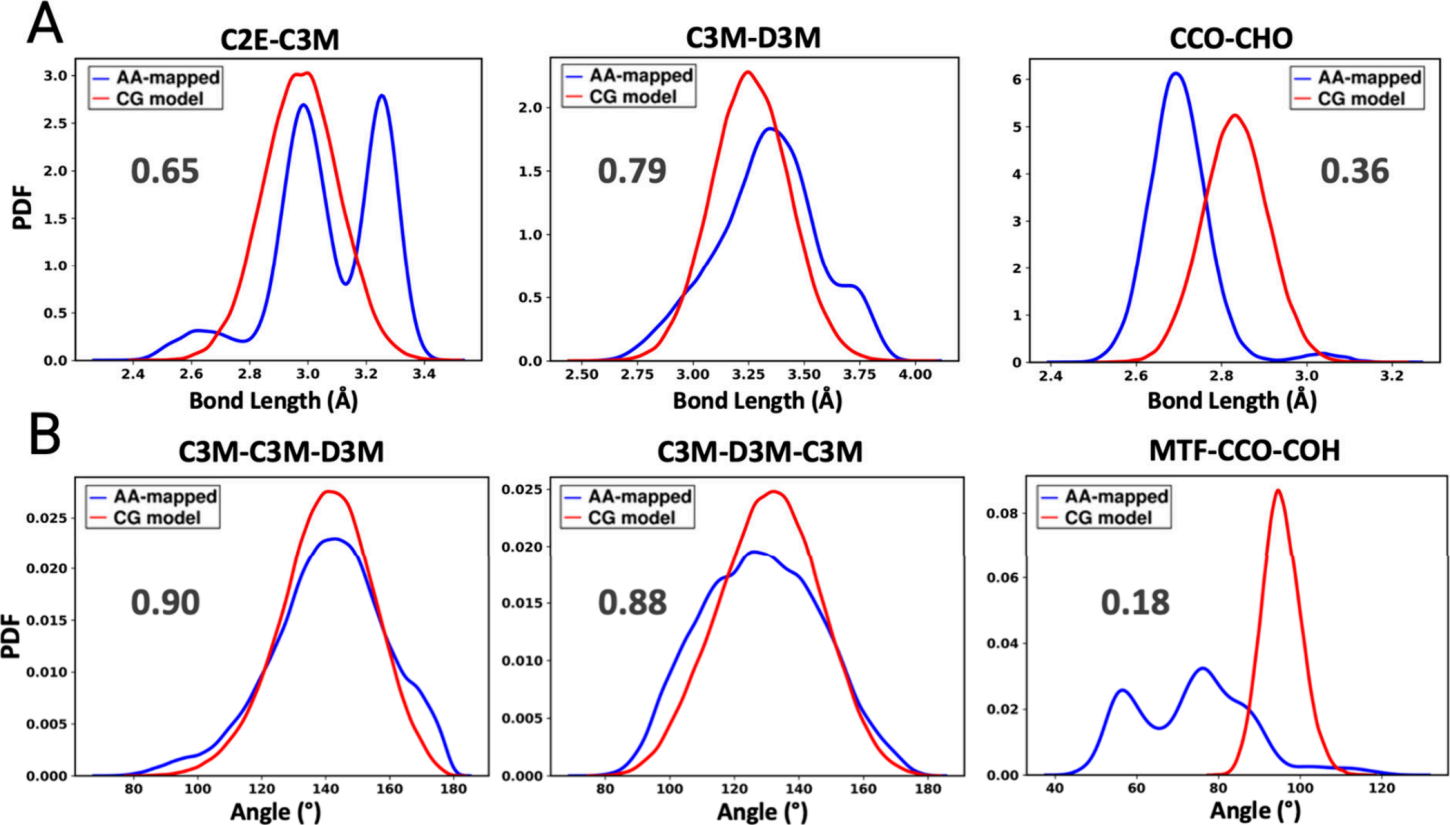
Representative probability density functions for **A)** bond lengths and **B)** angles for CG lipids (red) compared with mapped atomistic distributions (blue). Refer to Figure 1 for mapping scheme and bead definitions. A large overlap between the blue and red lines indicates good agreement between the local structure captured by the CG models and that obtained from the AA-mapped trajectories. This is quantified by the overlap coefficients, which are shown in bold black text on each plot. The shifted distributions observed in the CG models are attributed to the use of the “exclude 1–2” criterion in CG MD simulations.

Similar results are observed for the PDFs of angles in the optimized CG lipid models as shown in Figure 2B and Figure S6 in the SI. For example, the graphs shown in Figure S6 in the SI exhibit a reasonable overlap (OCs > 0.5) between the AA mapped distributions in terms of the mean values and variance (11 out of 16 distributions). PDFs of angles near the headgroup show narrowing or shifting from their desired values as seen in the MTF-CCO-COH angle in Figure 2B. These effects are identified to be a result of competing forces exerted by bonded and nonbonded neighbors on beads in the densely crowded head region of the lipid molecules (e.g., PO2-CCO-COH, PO2-CCO-MTF etc.) because of nonbonded interactions between 1 and 3 beads.^89^ Due to the “1–2” exclusion criterion utilized during CG MD simulations, beads connected through an angle potential (1–3 pairs) in the head region are found positioned close enough to experience van der Waals repulsive forces from one another. This interaction results in an overall shift in their distribution compared to AA mapped distributions, which is particularly observed between COH and MTF beads, where the spatial distance of 4.8 Å (as shown in Figure S9 in the SI) is less than σ_COH‑MTF_ (4.95 Å). As a result, lipids appear more elongated along the bilayer normal, increasing *D*_PP_ while maintaining *A*_L_ consistent with experiments. Simulations performed using the “1–3” exclusion scheme led to bond and angle distributions that more closely matched AA distributions but resulted in even lower *A*_L_ and larger *D*_PP_ values, highlighting the crucial influence of this factor on membrane properties. The competing effects between bonded and nonbonded interactions are not observed in the hydrocarbon chains due to their linear nature. Large angle values prevent 1–3 bead pairs from coming close enough to experience significant van der Waals’ repulsions. As shown in Figure S9 in the SI, the lowest distance between C3M beads (across the kink) is ∼5.5 Å, which is significantly greater than σ_C3M‑C3M_ (4.63 Å), resulting in mild attraction between the beads.

To confirm this nonbonded effect on bond and angle PDFs, we performed CG MD simulations on the optimized models using the “exclude 1–3” criterion, which negates the effect of nonbonded interaction up to third bonded neighbors. The deviated bond and angle PDFs now show better agreement with their AA mapped counterparts and display greater OCs following this change. These data are presented in Figure S7–S8 in the SI. However, it has been previously observed that the “1–3” exclusion criterion reduces the overall stability of CG MD simulations at higher time steps.^89^ Hence, although the “1–3” exclusion criterion provides a better structural representation of the lipids, we ultimately use the “1–2” criterion considering most bonds and angles show good agreement with mapped distributions.^84,86–91,98,99^ Moreover, the use of “1–2” criterion ensures that the lipid models are consistent with our previously developed CG models and could be used with higher time steps of 10 to 20 fs to model hybrid lipid systems.

Additionally, as can be seen from the C2E-C3M bond distribution, MTF-CCO-COH angle distribution, and several other bond and angle distributions in the SI, AA mapped distributions often show multiple peaks, suggesting multiple stable bond and angle configurations. These multiple peaks can be attributed to the coexistence of trans–gauche conformations in atomistic structures.^87,138^ However, in the context of CG distributions, these multiple peaks can not be reproduced considering the use of simple harmonic potential forms for bonds and angles.

#### 3.2.2 Radial Distribution Functions

Radial Distribution Functions (RDFs) between different CG beads in the lipid molecules are calculated using the CPPTRAJ package and compared with the averaged AA mapped data from all three lipids.^139^ These can be found in Figure S10 in the SI, with plots for the newly developed beads shown in Figure 3. The optimized CG models show good agreement with the averaged atomistic RDFs, albeit with some enhanced ordering observed in the CG model. Note that enhanced ordering and stronger structural correlations in CG models have been reported in previous studies.^84,86,140^ However, the positions of the first peaks match reasonably well for all CG beads since they are already considered for obtaining initial ranges while optimizing the nonbonded interaction parameters.

**Figure 3:**
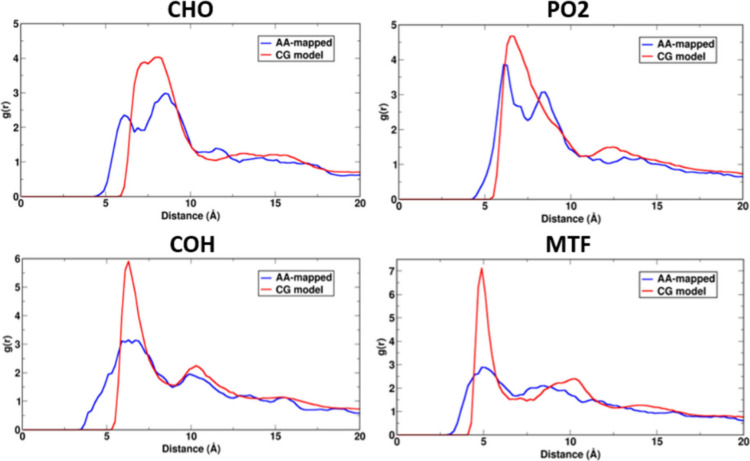
RDFs for newly developed CG lipid beads (red) compared with the RDFs from AA-mapped simulation trajectories (blue). Refer to Figure 1 for mapping schemes and bead definitions. CG models exhibit stronger structural correlation compared to mapped AA RDFs.

Additionally, the RDFs between new CG beads and water are calculated to investigate the structure of water around the bilayers as compared to atomistic-mapped RDFs (Figure S11 in the SI). AA water molecules are mapped onto their CG counterparts by identifying the closest pairs of water molecules, across the simulation box and calculating their centers of mass. The observed RDFs from CG models are slightly right-shifted, with more pronounced hydration peaks. It is worth noting that, even though the interaction parameters between lipid and water beads are not optimized to reproduce this structure, our models still capture some of the structural details at the lipid–water interface. Multiple prominent peaks are observed in RDFs for CHO-W and MTF-W, hinting at greater ordering of water around these beads as compared to beads PO2 and COH. We also notice that CG beads in DOPC displayed maximum hydration, followed by the POPC and DMPC systems.

### 3.3 Effects of CG MD Simulation Variables

Several CG models have been reported to exhibit changes in properties, when variables like the system size^86,141^ or the simulation time step^142,143^ are altered. Hence, to systematically ascertain the robustness of our newly developed models to these variables, multiple 200 ns simulations with varying system sizes and time steps are performed and analyzed according to the aforementioned strategy.

#### 3.3.1 Effect of System Size

The effect of system size is studied by simulating DOPC, POPC, and DMPC bilayers with 128, 288, and 576 lipids using a simulation time step of 10 fs. The AA initial configurations for the 288, and 576 lipid systems are obtained from the Charmm-GUI package, coarse grained, and solvated using 1-site CG water beads as described in Section 2.4.2.^86^ Table 3 presents the data obtained from these systems, which show that the system size does not have any significant effect on the calculated properties indicating the robustness of our models. The smallest differences are observed for the *D*_PP_ values, while *A*_L_ and κ show some variations but are still in good agreement with mean simulated properties. Additionally, as expected, the standard deviation is found to decrease as the system sizes increase. The numerical data for these results can be found in **Tables S3–S5** in the SI.

**Table 3:** Structural and Elastic Properties of DOPC, POPC and DMPC Simulated with Different System Sizes (128, 288, and 576 Lipids)^a^

	DOPC (298 K)			POPC (298 K)			DMPC (317 K)		
System size	*A*_L_ (Å^2^)	*D*_PP_ (Å)	κ (*k*_B_ *T*)	*A*_L_ (Å^2^)	*D*_PP_ (Å)	κ (*k*_B_ *T*)	*A*_L_ (Å^2^)	*D*_PP_ (Å)	κ (*k*_B_ *T*)
128 lipids	66.95 ± 1.4	39.78 ± 0.9	22.0 ± 1.5	65.28 ± 1.4	39.2 ± 0.9	26.08 ± 1.3	64.8 ± 1.6	34.43 ± 0.8	27.15 ± 1.5
288 lipids	65.65 ± 1.2	40.03 ± 0.6	24.10 ± 0.9	65.53 ± 1.0	39.18 ± 0.6	25.93 ± 0.8	64.73 ± 1.3	34.50 ± 0.6	26.25 ± 0.9
576 lipids	65.70 ± 1.0	40.00 ± 0.6	22.43 ± 0.7	65.23 ± 0.8	39.38 ± 0.5	25.43 ± 0.7	64.70 ± 0.9	34.55 ± 0.4	26.50 ± 0.9

a Values presented were obtained from 200 ns simulations using the protocol specified in Section 2.5.

#### 3.3.2 Effect of Simulation Time Step

The 128-lipid systems for DOPC, POPC, and DMPC used during parameter optimization are next simulated for 200 ns with time steps of 5, 10, 15, 20, and 25 fs to investigate the effect of simulation time step as presented in Table 4 and Tables S6–S8 in the SI. The change in simulation time steps does not produce any major differences in the bilayer properties where all calculated properties are comparable to our experimental and mean simulated values.

**Table 4:** Structural and Elastic Properties of Simulated DOPC, POPC and DMPC Systems Using Different Simulation Time Steps^a^

	DOPC (298 K)			POPC (298 K)			DMPC (317 K)		
Time step	*A*_L_ (Å^2^)	*D*_PP_ (Å)	κ (*k*_B_ *T*)	*A*_L_ (Å^2^)	*D*_PP_ (Å)	κ (*k*_B_ *T*)	*A*_L_ (Å^2^)	*D*_PP_ (Å)	κ (*k*_B_ *T*)
5 fs	65.5 ± 1.3	39.8 ± 0.9	23.2 ± 1.5	65.3 ± 1.2	39.3 ± 0.8	25.2 ± 1.2	64.6 ± 1.7	34.2 ± 0.8	26.9 ± 1.3
10 fs	66.95 ± 1.4	39.78 ± 0.9	22.0 ± 1.5	65.28 ± 1.4	39.2 ± 0.9	26.08 ± 1.3	64.8 ± 1.6	34.43 ± 0.8	27.15 ± 1.5
15 fs	65.5 ± 1.6	39.5 ± 0.9	24.1 ± 1.3	64.9 ± 1.3	39.0 ± 0.8	27.3 ± 1.3	64.6 ± 1.7	34.2 ± 0.9	25.7 ± 1.2
20 fs	66.4 ± 1.7	39.4 ± 0.8	23.3 ± 1.4	65.4 ± 1.7	38.6 ± 0.9	26.7 ± 1.3	64.8 ± 1.9	34.0 ± 0.8	25.9 ± 1.4
25 fs	66.4 ± 1.5	39.6 ± 0.9	23.3 ± 1.5	65.1 ± 1.3	38.9 ± 0.8	25.8 ± 1.1	64.3 ± 1.7	34.0 ± 0.8	27.4 ± 1.6

a Values presented were obtained from 200 ns simulations using the protocol specified in Section 2.5.

### 3.4 Sensitivity Analysis

To further understand the complex relationships between interaction parameters and the resultant simulated properties, we perform sensitivity analysis of our models. Using a Sobol sequence, 5000 additional parameter sets are generated within a 5% range around the optimized parameter values and CG MD simulations are carried out using these perturbed parameter sets. The 5000 perturbed parameter sets, along with their corresponding bilayer properties (calculated for the final 5 ns out of a total 25 ns run time) are processed to screen outliers beyond three standard deviations from the mean, and a final database of 4653 entries is obtained.

#### 3.4.1 Coefficients of Variation

The distributions of properties obtained from these 4653 simulations are plotted as shown in Figure 4A. The coefficient of variation (CoV) is calculated for these distributions, as the ratio of standard deviation to the mean, to obtain a normalized measure of the dispersion of properties. Expressed as percentages, the CoVs for *A*_L_ (DOPC: 3.2%, POPC: 3.3%, and DMPC: 3.5%), and *D*_PP_ (DOPC: 2%, POPC: 2.1%, and DMPC: 2.1%) are found to be much lower than the 5% perturbation used for parameters, indicating that these properties are very robust to the parameters used. On the other hand, CoVs for κ (DOPC: 15.7%, POPC: 17.1%, and DMPC: 13.5%) are greater than 5%, indicating that κ values are sensitive to the parameters used. Additionally, by comparing the property distributions from these simulations with the mean simulated values we find that most simulated means lay close to the peaks of their respective distributions, indicating that the majority of perturbed parameters result in properties comparable to the optimized set. Notable exceptions to this are the distributions of κ which demonstrate that our models are more robust toward *A*_L_ and *D*_PP_ compared to κ. This is consistent with the data shown in Table 1.

**Figure 4:**
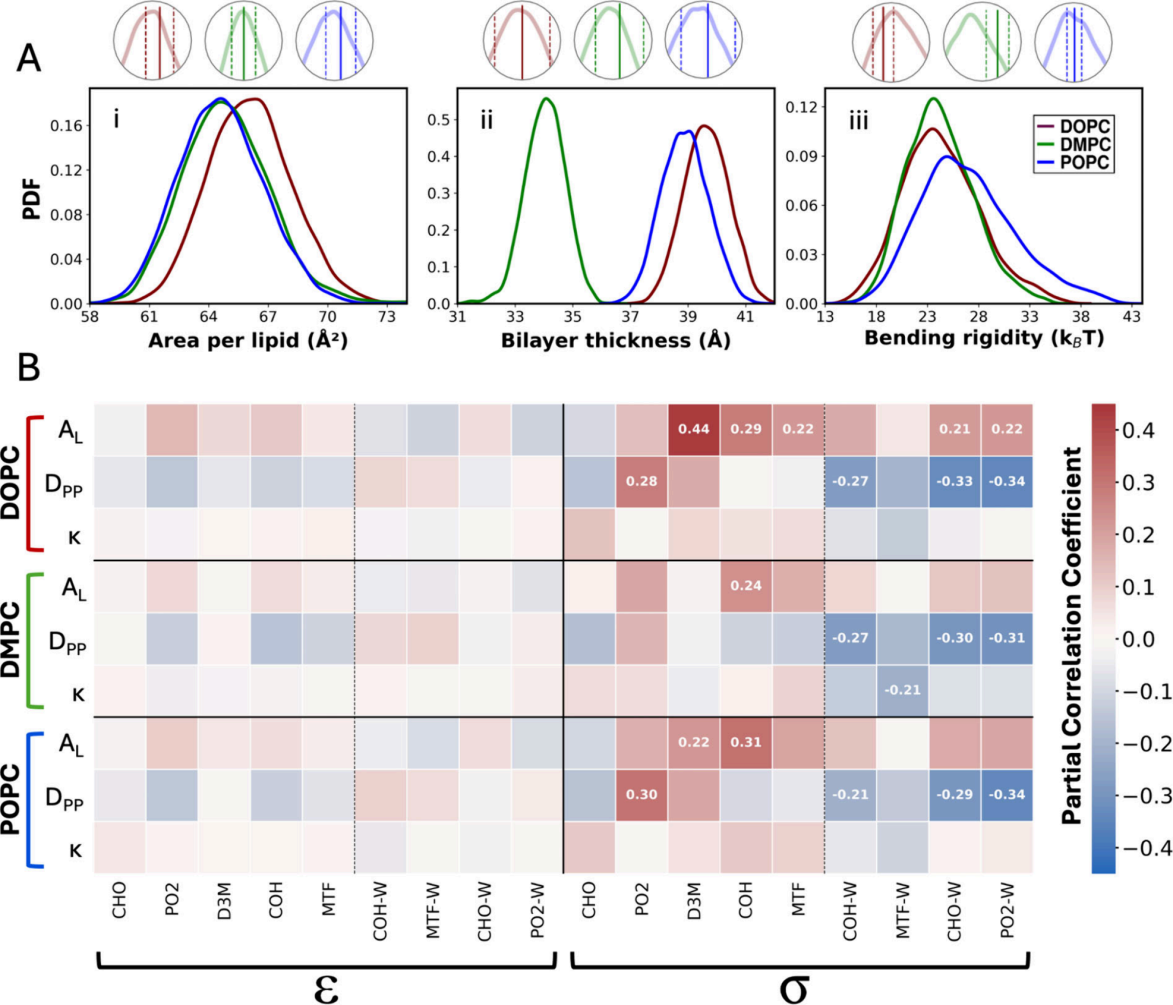
**A)** PDFs of bilayer properties (i) *A*_L_, (ii) *D*_PP_, and (iii) κ calculated from the 4653 perturbed parameter sets. Legend for the colors is provided in (iii). Inset images show individual PDF peaks obtained from these 4653 simulations along with the mean value (solid lines) and standard deviation (dashed lines) shown in Table 1 for the original optimized parameters. The peaks for most properties coincide with the mean values indicating low deviations in properties. **B)** Heat map showing the partial correlation coefficients (PCCs) between perturbed parameters and the calculated bilayer properties. Only squares with absolute PCCs > 0.2 show their respective annotated values. The color bar for the heat map is shown on the right where −0.4 and +0.4 show weakest and strongest correlations observed, respectively. Refer to Figure 1 for mapping schemes, bead types, and their locations.

#### 3.4.2 Partial Correlation Coefficients

Partial correlation coefficients (PCCs) are calculated using a Python script to assess the direct relationship between individual model parameters and calculated properties, while controlling for the influence of other parameters.^144,145^ This involves fitting linear models to isolate residuals of each parameter and property from other covariates, and then computing the Pearson correlation between these residuals. This approach allows for a precise evaluation of the contribution of each parameter to properties independent of other variables, providing a clear understanding of the direct effects in the data.^144,145^ PCC values range from −1 to 1, where values closer to these limits indicate strong linear relationships between variables while values near 0 suggest little to no linear relationship. Figure 4B presents a heatmap of PCCs calculated between all parameters and properties involved during the model development process. It suggests that all *ε* parameters show absolute PCCs < 0.2 indicating that the perturbations within 5% of original *ε* values do not directly and significantly impact the model’s prediction of properties. In case of *σ* parameters, however, impact of perturbing parameters is observed. Most *σ* parameters exhibit higher positive correlation with the calculated *A*_L_ values, which means that as these parameters are increased, the *A*_L_ of the simulated bilayers increases, indicating sensitivity toward *σ* perturbation. Given that *σ* values govern the distances between interacting nonbonded beads, this observation is to be expected. On the other hand, *D*_PP_ values in all lipid molecules are discovered to be negatively correlated to the cross-interaction σ parameters, specifically *σ*_COH‑W_, *σ*_CHO‑W_, and *σ*_PO2‑W_, with similar PCC values between −0.34 and −0.29. This means that as values for these parameters are increased, *D*_PP_ values decrease, as one would expect given the trends in *A*_L_. This may also explain the right-shift observed in hydration RDFs, where due to the flexibility in optimization afforded to *σ*_headgroup‑water_ parameters, *σ* values for our models are larger to reduce the errors with the calculated *D*_PP_ values. Larger *σ*_headgroup‑water_ values cause water beads between adjacent lipid head beads to push these headgroups further apart, resulting in weaker packing and lower *D*_PP_. Thus, while *A*_L_ values are most sensitive to self-interaction *σ*, *D*_PP_ values are sensitive to cross-interaction *σ* parameters in our model. Interestingly, κ values for all three lipid types do not show notable direct impact of specific parameters, but still show the highest CoVs, indicating higher inherent uncertainty in κ calculations from CG MD simulations. To further assess the applicability of our models in capturing membrane elasticity, we compute the area compressibility modulus (*K*_A_) from *A*_L_ fluctuations in the CG simulations, following the method described by Pluhakova et al.,^131^ and many others.^33,146,147^ The *K*_A_ values obtained from CG simulations for DOPC, POPC, and DMPC are 381.5, 428.2, and 306.1 mN/m, respectively. We also utilized an alternative method to calculate *K*_A_ from real-space analysis of local thickness fluctuations presented by Doktorova et al.,^32,37,113^ which yielded values of 342.7, 357.1, and 336.8 mN/m, respectively. These values fall within the broad ranges reported for *K*_A_ from other models in literature (DOPC: 251–840 mN/m;^32,37,113^ POPC: 353–861 mN/m;^131^ DMPC: 287–840 mN/m^131^). Values obtained using both approaches, however, remain slightly overestimated relative to the reported experimental measurements (DOPC: 265–310 mN/m, POPC: 180–330 mN/m, DMPC: 145–234 mN/m),^148–151^ consistent with previous reports attributing such discrepancies to underestimated area fluctuations during simulations.^37,131,152^ These results nevertheless support our models’ ability to reasonably capture additional elastic properties beyond bending rigidity.

### 3.5 Self-Assembly Studies

The organization of lipids into specific assemblies depending on factors such as their structural characteristics, temperature, or concentration is an important feature of these molecules and is responsible for most of their interesting functions.^153^ It is hence essential for lipid CG models to satisfactorily capture this self-assembly process. Having only studied preassembled bilayers so far, we next evaluate our models’ capability to reproduce self-assemblies. Several mixed lipid–water systems with DOPC, POPC, and DMPC are created using the PACKMOL package and simulated using the NPT ensemble for 200 ns to probe their self-assembly into bilayers or vesicles.^154^ Most of the simulation protocols described in Section 2.4.2 are kept unchanged for these simulations.

#### 3.5.1 Self-Assembly of Bilayers

Randomly mixed systems containing 128 lipids and 7645 water beads in a cuboidal box of dimensions ∼65 Å × 65 Å × 120 Å (same as the box size with preassembled bilayers used for parameter optimization) are used to study the self-assembly of bilayers as shown in Figure 5A. The randomly dispersed lipid molecules are observed to spontaneously assemble into a continuous phase within the initial 20 ns of the simulations for all tested lipid types DOPC, POPC, and DMPC. The self-assembly process for DOPC bilayer is presented as a series of snapshots in Figure 5B, which shows the lipid molecules coalescing into a continuous fiber-like phase due to the cuboidal box used in the simulation, which eventually interacts with its periodic image to form a bilayer along the cross-section of the simulation box.^60^ The formation of a lipid bridge is observed during the initial stages of lipid aggregation which breaks apart within 5 ns. A similar mechanism of spontaneous bilayer formation is observed for POPC and DMPC bilayers and has also been previously reported in literature.^60^ The assembled bilayers also show good agreement with the simulated *A*_L_ and *D*_PP_ values obtained from preassembled bilayers as well as experimental data.

**Figure 5:**
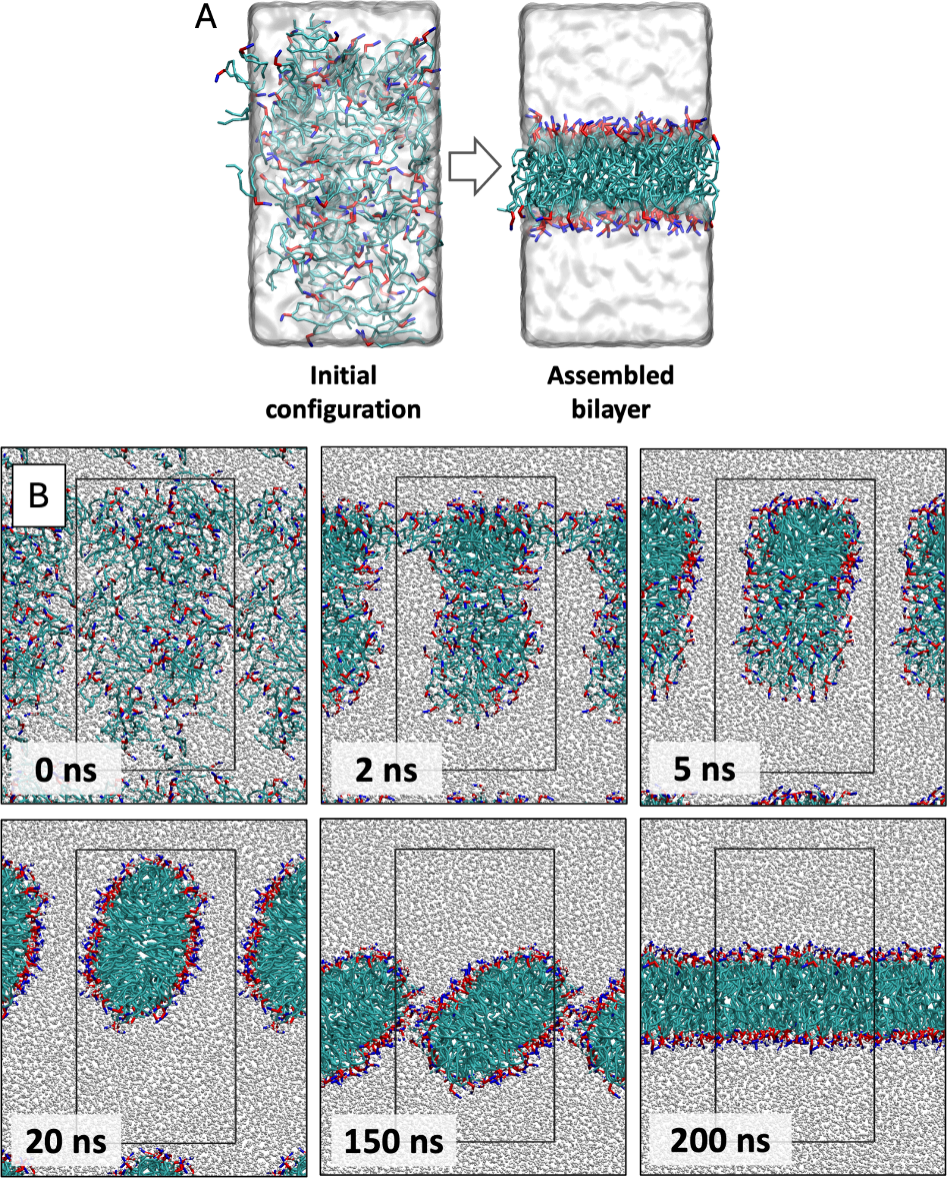
Representative examples from self-assembly simulations for DOPC lipids. **A)** Snapshots of initial and final configurations show the assembly of lipid molecules into a bilayer. **B)** Snapshots showing the self-assembly of bilayers in DOPC simulations. The black rectangle represents the periodic boundaries and water is represented as gray spheres. The color scheme is consistent with initial configuration snapshots in Figure 1.

#### 3.5.2 Self-Assembly of Vesicles

Systems containing 1024 lipids concentrated within a box of 300,000 water beads having dimensions ∼275 Å × 275 Å × 275 Å are used to observe the self-assembly of vesicles. Figure 6A shows the initial configuration for these simulations, which is chosen to prevent lipid molecules from interacting with other lipids across the periodic boundaries, in order to facilitate the formation of a single vesicle. Self-assembled vesicles are observed in as little as 30 ns with final snapshots for the DOPC vesicle as shown in Figure 6A. Aggregation of lipid molecules into locally formed bicelle-like structures is observed at the beginning of simulations.^155^ The aggregates coalesce into a single phase with curved edges as shown in Figure 6A. Similar behavior has been previously observed by Marrink et al.,^155^ where they state that the line tension is minimized by encapsulating water to form a cup-like structure with a small pore. Additionally, Figure S12 in the SI presents snapshots exhibiting the sealing of the vesicle pore where lipid molecules near the surface of the pore change their orientations as they aggregate together. Figure 6C and S13 in the SI show the normalized distributions of several CG beads with respect to the center of the vesicle. Note, Figure 6B shows a schematic of the distance from the vesicle center to its surface. The plots clearly show a bilayered structure of the vesicle with no lipid beads closer than 15 Å from the center of the vesicle. The asymmetric peaks for CHO and PO2 beads indicate a tighter packed state of lipid headgroups in the inner leaflet, i.e. closer to the vesicle core. The vesicle inner radii are found to be ∼25–30 Å, based on the inner distribution peak for the CHO beads. Similarly, the *D*_PP_ value for the assembled vesicles, estimated based on the distribution peaks for PO2 beads is found to be ∼35–40 Å, which is in agreement with bilayer *D*_PP_ values.^155^ The distribution of water beads shown in blue indicates that the interior cavity is filled with water (0 Å to 20 Å), with a bulk water phase located beyond the outer radius of the self-assembled vesicles (>90 Å). As expected, no water molecules are observed within regions occupied by the hydrocarbon chains of the vesicle (45 Å to 65 Å). Our observations agree with previously published results from vesicle studies highlighting the capabilities of our models in generating self-assembled lipid structures.^155–158^ Here, we note that the self-assembly of bilayers and vesicles in our CG simulations occurs within tens of nanoseconds, which is significantly faster than in atomistic simulations or experiments. While CG models are known to accelerate dynamics by 1–2 orders of magnitude due to reduced friction and smoother potential energy surfaces,^60,159^ rapid assembly observed here likely also reflects the efficiency of our force field parametrization, especially the treatment of polar and nonpolar interactions. Importantly, the observed assembly pathway remains consistent with experimental and atomistic findings, supporting the physical relevance of the approach.^155^

**Figure 6:**
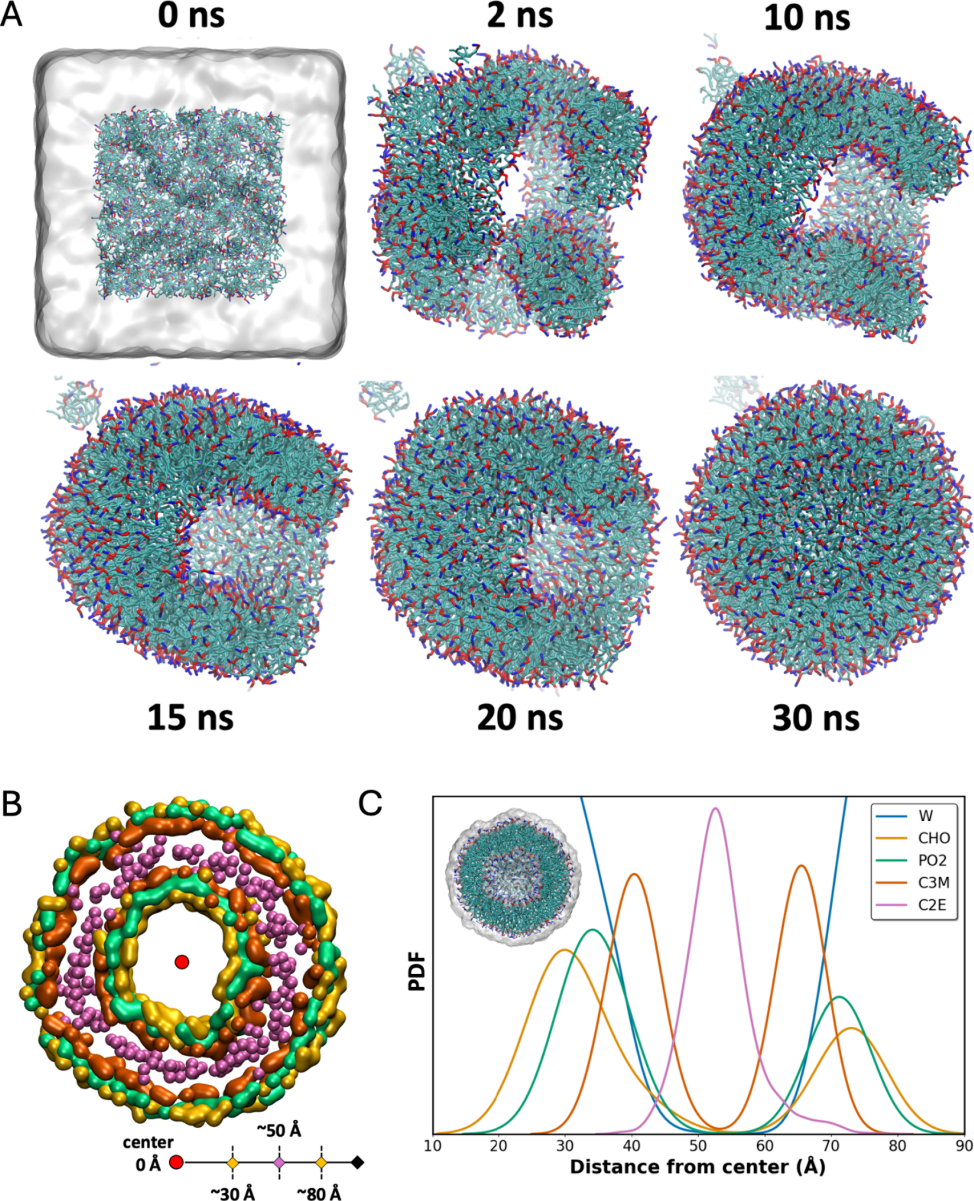
**A)** Snapshots showing the self-assembly of a DOPC vesicle. Water is hidden after the initial configuration for convenience of visualization. **B)** Representative snapshot showing the cross section of a central 20 Å-thick slice of the assembled vesicle. Note that the vesicle interior contains water beads which are hidden for clarity. **C)** Normalized PDFs of different lipid beads assembled into a vesicle: CHO, PO2, first C3M (closest to the glycerol backbone), and C2E (at the end of tail groups) beads, with respect to the center of the assembled vesicle. Snapshot in **B** shows the same beads and is also colored according to the legend presented in plot **C**. Normalized PDF of water beads is also shown in blue which suggests that no water was present in the hydrocarbon-rich regions. Inset shows the cross-section view of the assembled vesicle, consistent with **A**. Here, neighboring and enclosed water is shown as a white semitransparent surface for visual reference.

### 3.6 Test of Transferability

Finally, to test the chemical transferability of our CG models, MD simulations are performed on four other lipid molecules not utilized during parameter optimization. Specifically, we modeled 1,2-dilauroyl-sn-glycero-3-phosphocholine (DLPC), 1,2-dipalmitoyl-sn-glycero-3-phosphocholine (DPPC), 1,2-distearoyl-sn-glycero-3-phosphocholine (DSPC), and 1-stearoyl-2-oleoyl-sn-glycero-3-phosphocholine (SOPC). These lipid molecules, which can be modeled using the same CG beads developed in this study (Figure S14), chemically differ from the lipids used for FF optimization. Specifically, DLPC, DPPC, and DSPC are fully saturated lipids with increasing hydrocarbon chain lengths of 12, 16, and 18 carbons, respectively. On the other hand, SOPC contains an 18:0 saturated tail and an 18:1 unsaturated tail, introducing both chemical and length asymmetry across the two hydrocarbon chains of the lipid. These distinctions in chain length, saturation, and asymmetry reflect nontrivial differences in the underlying chain structure, enabling a rigorous evaluation of the model’s chemical transferability. 128-lipid preassembled bilayer systems are obtained from CHARMM-GUI and coarse-grained to obtain initial configurations for our simulations. CG MD simulations are performed using the optimized model parameters and the calculated properties are compared with data from published literature.^41,129,160–162^ While SOPC and DLPC systems are simulated at a temperature of 303 K, DPPC and DSPC membranes are simulated at a temperature of 323 and 333 K, respectively, owing to their high transition temperatures (∼313 K and ∼328 K, respectively). This additionally allows us to examine the performance of our models at much higher temperatures than were used for model development. Table 5 shows the calculated lipid properties for different system sizes and simulation times for these validation simulations. The simulated systems show good agreement with *A*_L_ and *D*_PP_ values from previous studies and acceptable agreement with the bending rigidity values, indicating the chemical transferability of our CG beads in simulating a variety of lipid systems. The bending rigidity for the DSPC system simulated at 333 K shows a deviation of ∼20 *k*_B_ *T* from the literature values, followed by the DPPC system (323 K) which deviates ∼8 *k*_B_ *T* suggesting that our models may require additional tuning for bending rigidity calculations at temperatures beyond 320 K.

**Table 5:** Structural and Elastic Properties of DLPC, DPPC, DSPC, and SOPC Bilayers Simulated Using Our Optimized Interaction Parameters^a^

	**DLPC** (303 K)			**DPPC** (323 K)			**DSPC** (333 K)			**SOPC** (303 K)		
128 lipid system	***A***_**L**_	***D***_**PP**_	**κ**	***A***_**L**_	***D***_**PP**_	**κ**	***A***_**L**_	***D***_**PP**_	**κ**	***A***_**L**_	***D***_**PP**_	**κ**
**Exp**	60.8 ^129^	29.8 ^129^	-	63.1 ^129^	38.6 ^129^	35.0 ^160^	63.8 ^129^	42.2 ^129^	∼42 ^19^	65.5 ^129^	38.6 ^129^	21.4 ^161^
**AA MD**	62.8 ^41^	-	25.8 ^41^	61.2 ^41^	-	34.1 ^41^	61.8 ^162^	44.2 ^162^	-	63.8 ^41^	-	26.4 ^41^
40–50 ns	60.8 ± 0.5	31.4 ± 0.4	30.7 ± 0.8	65.4 ± 0.8	37.3 ± 0.5	23.5 ± 0.7	65.2 ± 0.7	40.2 ± 0.5	21.0 ± 0.9	66.0 ± 0.5	40.8 ± 0.4	26.4 ± 0.7
90–100 ns	61.5 ± 0.7	31.6 ± 0.3	31.7 ± 0.6	66.2 ± 0.6	37.0 ± 0.5	26.0 ± 0.6	66.9 ± 0.7	40.3 ± 0.4	21.8 ± 0.8	65.5 ± 0.8	40.8 ± 0.5	24.0 ± 0.8
140–150 ns	62.9 ± 0.8	31.4 ± 0.4	26.5 ± 0.5	65.1 ± 0.6	37.1 ± 0.3	27.8 ± 0.7	67.9 ± 0.7	40.4 ± 0.7	24.1 ± 1.1	64.7 ± 0.7	40.6 ± 0.3	23.9 ± 0.6
190–200 ns	62.7 ± 0.8	31.5 ± 0.4	24.1 ± 0.6	64.8 ± 0.7	37.1 ± 0.4	30.2 ± 0.8	71.2 ± 0.7	39.4 ± 0.6	20.1 ± 0.9	64.3 ± 0.7	40.7 ± 0.5	25.6 ± 0.6
**Mean**	62.0 ± 1.4	31.5 ± 0.8	28.3 ± 1.3	65.4 ± 1.4	37.1 ± 0.9	26.9 ± 0.9	67.8 ± 1.4	40.1 ± 1.1	21.8 ± 1.9	65.1 ± 1.4	40.7 ± 0.9	25.0 ± 1.4

a *A*_L_ values are presented in units of Å^2^, *D*_PP_ values are presented in Å, whereas κ values are presented in *k*_B_ *T* units.

To further evaluate the temperature transferability of our CG lipid model, we simulated membranes of saturated PC lipids—namely DLPC, DMPC, DPPC, and DSPC—across a range of temperatures (303 to 353 K) using our optimized CG interaction parameters. These simulations are initialized from fluid-phase bilayers following the same simulation protocols presented in Section 2.4. The hydrophobic thickness (2*D*_C_) and *A*_L_ are calculated and compared to reference AA data reported by Zhuang et al.^162^ As shown in Table S9, the CG models reproduce the expected temperature dependence of membrane structures across all lipid types and show qualitative agreement with AA simulations.^162^ The following trends are observed: (*i*) Hydrophobic thickness decreases with temperature (indicative of chain disorder), and (*ii*) *A*_L_ increases with temperature (indicative of bilayer expansion).^129,162,163^ Thus, the observed temperature trends confirm the ability of our CG model to predict membrane behavior across a wide temperature range without reparametrization. While simulations below transition temperatures do not result in full gel phase transitions, such transitions are known to occur on much longer time scales or require nucleated initiation which will be explored in detail in future studies.^64,164–166^

## 4 Conclusions

The developed CG models in this work offer several notable advantages in simulating lipid bilayers. First, the models exhibit strong consistency in predicting key structural properties such as area per lipid (*A*_L_), bilayer thickness (*D*_PP_), as well as elastic or mechanical properties such as the bending modulus (κ) across various lipid types. This consistency is achieved while maintaining computational efficiency, allowing exploration of large length scale (systems with millions of atoms) and longer time scale (up to several microseconds), which is otherwise challenging to study using atomistic models. The models exhibit good chemical transferability, successfully extending to lipid molecules not included in the original parameter optimization process, which highlights its versatility and applicability to a wide range of lipid systems. The models demonstrate feasibility in simulating self-assembly processes, such as the formation of bilayers and vesicles. A unique aspect of our models is the systematic tuning of interaction parameters ensuring that they can be easily adapted or refined for specific research needs. By systematically optimizing the force field against both structural and elastic experimental data obtained from SAXS/SANS and NSE, our models provide a comprehensive and physically grounded description of membrane behavior which complements existing CG lipid models that primarily or solely target structural properties. This enables accurate modeling of equilibrium structures, mechanical responses, and self-assembly while maintaining transferability to chemically distinct lipid types and hybrid systems. These features distinguish our models as versatile and robust tools for studying complex membrane systems at mesoscopic scales.

However, we acknowledge some limitations that are characteristic of coarse-grained modeling, with opportunities for further refinement. For instance, the simplified potential form in our CG models results in minor discrepancies in bond and angle distributions, especially near lipid headgroups. These behaviors are expected given the resolution of CG models and do not necessarily impact their structural accuracy or chemical transferability. Future refinement of our models could include the use of advanced bonded potentials, mixed-resolution strategies, or broader training sets for parameter optimization, which may mitigate some of these discrepancies. Still, even in their current form, our models successfully replicate key membrane properties and demonstrate chemical transferability across different zwitterionic lipids, enabling studies of complex lipid assemblies and their behavior under various conditions. Notably, these models could be used to simulate more complex structures such as lipid mixtures, lipid–polymer hybrids, and lipid-glycomaterials systems. We also note that while our present models lack explicit electrostatic interactions, they are well-suited for studying a number of biologically and technologically relevant lipid systems where hydrophobic and steric interactions dominate. These include hybrid lipid–polymer membranes used in designing polymer-stabilized liposomes,^167^ synthetic PC lipid membranes with tailored mechanical properties,^168^ and the interaction of membranes with uncharged nanoparticles,polymers, or glycomaterials.^169^ Such applications profoundly benefit from CG modeling,^53,55,94–96^ thus structurally and elastically optimized models like the ones developed in this work offer advanced capabilities for guiding materials design and predicting functional performance.

## Supplementary Material



## Data Availability

NAMD topology and parameter files for the developed CG models as well as several analysis codes used in this paper are available to access using the following GitHub link: https://github.com/Deshmukh-Group/CG-Lipid-model. Any additional data from the current study are available from the corresponding author upon reasonable request.

## Notes

Experimental methods for NSE, SAXS, and SANS measurements; details of the PSO optimization framework; complete CG force field parameters and mapping schemes; additional validation of structural and elastic bilayer properties; bond, angle, and radial distribution analyses; tabular data for model validation using different simulation variables and self-assembly snapshots; and topology and parameter files for the developed CG lipid models (PDF)
