# Supplementary material for: Development of Transferable Coarse-Grained Lipid Models with Optimized Structural and Elastic Membrane Properties
Source: J Chem Theory Comput. 2025 Sep 23;21(19):9890–908. doi: 10.1021/acs.jctc.5c00579 (PMC12529907; doi:10.1021/acs.jctc.5c00579)
Supplement: Supplementary file 1 [file ct5c00579_si_001.pdf]

Supporting Information

Development of Transferable Coarse-Grained Lipid Models with Optimized Structural and Elastic Membrane Properties

Soumil Y. Joshi[1], Teshani Kumarage[2], Rana Ashkar[2]*, and Sanket A. Deshmukh[1]*

[1]*Department of Chemical Engineering, Virginia Tech, Blacksburg, VA 24061, USA*
[2]*Department of Physics and Center for Soft Matter and Biological Physics, Virginia Tech, Blacksburg, VA 24061, USA*

*Corresponding Authors:
Sanket A. Deshmukh (sanketad@vt.edu), Rana Ashkar (ashkar@vt.edu)

S1. Sample preparation for NSE, SAXS and SANS experiments

Phospholipids were purchased from Avanti Polar Lipids (Albaster, AL) as dry powders and used without further purification. D_2O (99.9%) was purchased from Cambridge Isotope Laboratories, Inc. Filtered and purified H_2O was obtained using a Millipore Milli-Q water purification system with Direct-Q® 3 UV Kit. Lipid membranes were prepared in the form of unilamellar vesicles (ULVs) for SANS, SAXS, and NSE experiments using standard protocols,[1] as explained below.

First the required amount of lipids powder was dissolved in chloroform along with 4 mol% of charged lipids in order to avoid multilamellar stack formation.[2] Then the solvent was evaporated using a nitrogen gas stream followed by overnight vacuum drying at 35-37 °C in order to remove traces of any residual organic solvent. The buffer solution was prepared using D_2O for neutron scattering experiments (i.e. SANS and NSE) whereas H_2O was used for X-ray scattering experiments (i.e. SAXS). The dried thin films were then hydrated using the prepared buffer solutions and the samples were subjected to at least 5 freeze-thaw cycles using -80 °C freezer and 40 °C warm water bath with intermittent vortex mixing. The suspension was then extruded by performing 31 passes through 100 nm pore size polycarbonate filters using an automated home-built mini-extruder.[1] Liposomal suspensions for SAXS/SANS measurements were prepared at a total lipid concentration of 20 mg/mL, whereas NSE samples were prepared at a total lipid concentration of 50 mg/mL.

SAXS/SANS measurements

SAXS measurements were primarily carried out at ORNL using a Rigaku BioSAXS-2000 system (Rigaku Americas). This instrument features an HF007 copper rotating anode, a Pilatus 100K two-dimensional (2D) detector, and an automatic sample changer. Additional measurements were done on the XEUSS 3.0 SAXS instrument at Virginia Tech, using 1.5 nm

capillary cells. SAXS data were collected at a fixed sample-to-detector distance, calibrated by a silver behenate standard, with a typical data-collection time of 3 h. The one-dimensional (1D) scattering intensity was obtained by radial averaging of the corrected 2D detector images, after background subtraction, using the instrument software. All experiments were run on 20mg/mL ULV suspensions at 25 °C except for DMPC which was run at 30 and 44 °C. The latter is due to the higher melting transition of DMPC (~24°C), necessitating higher temperature runs to ensure that the membrane is in the fluid phase.

SANS experiments were performed at the NGB-30m SANS instrument at the National Institute of Standards and Technology (NIST) Center for Neutron Research (NCNR) on protiated vesicle suspensions of DOPC and chain deuterated vesicle suspensions of DMPC-d_{54}, and POPC-d_{31}. SANS data were collected over a range of ~0.001 to 0.5 $Å^{-1}$. All measurements were conducted using 1-mm path-length quartz banjo cells (Hellma USA, Plainview, NY) on 20 mg/mL ULV suspensions at 25 °C except for the DMPC sample which was run at 30 and 44 °C.

Here, we note that the scattering vector transfer, q is given by, $q = \frac{4\pi}{\lambda} sin(\theta)$, where λ is the neutron or X-ray wavelength and θ is the scattering angle relative to the incident beam. Similar to SAXS, the 1D SANS signals were obtained from radial averaging of the 2D scattering signals after correcting for resolution, empty cell scattering, and background. The 1D SAXS/SANS data were analyzed using SasView software[3] in accord with a modified scattering density profile model with 5 layers, as described in prior studies.[4]

Neutron Spin Echo measurements

NSE experiments were performed on vesicle suspensions as described above, i.e. 100 nm vesicles at 50 mg/mL lipid concentration. All measurements were conducted at 25 °C, except for the DMPC sample which was run at 30 °C and 44 °C, i.e. in the fluid phase above the melting temperature of DMPC. NSE experiments on DMPC, POPC and DOPC vesicle suspensions were performed on NSE spectrometer at the NIST Center for Neutron Research (NCNR) over a q-range of 0.04-0.1 $Å^{-1}$. The samples were preloaded in quartz cells with a path length of 2 mm and measured for 23 hrs each. Instrument resolution and buffer runs were collected under the same configurations as the measured samples for background subtraction and data normalization. Data reduction of NSE signals was performed using the Data Analysis and Visualization Environment (DAVE) software developed at NIST.[5]

This approach measures membrane dynamics in the form of decays of the normalized intermediate scattering function in terms of the Fourier time t. For membrane bending fluctuations, the observed decays follow a stretched exponential function given by the equation (**Eq. S1**):[6]

$$\frac{I\,(q,t)}{I\,(q,0)} = exp\left[-(\Gamma(q)t)^{\frac{2}{3}}\right] \qquad \text{...... } \textbf{\textit{Equation S1}}$$

where q is the scattering wave vector transfer defined as $q = \frac{4\pi}{\lambda}sin(\theta)$, with λ being the neutron wavelength. The rate of decay of membrane relaxation, $\Gamma(q)$, is directly proportional to the apparent membrane bending rigidity (κ) as expressed in **Eq. S2**, where η is the solvent viscosity, k_B is the Boltzmann constant, and T is the temperature on the absolute scale.[6–9]

$$\Gamma(q) = 0.0069\,\frac{k_BT}{\eta_{solvent}}\sqrt{\frac{k_BT}{\kappa}}\,q^3 \qquad \text{...... } \textbf{\textit{Equation S2}}$$

S2. Particle Swarm Optimization (PSO)

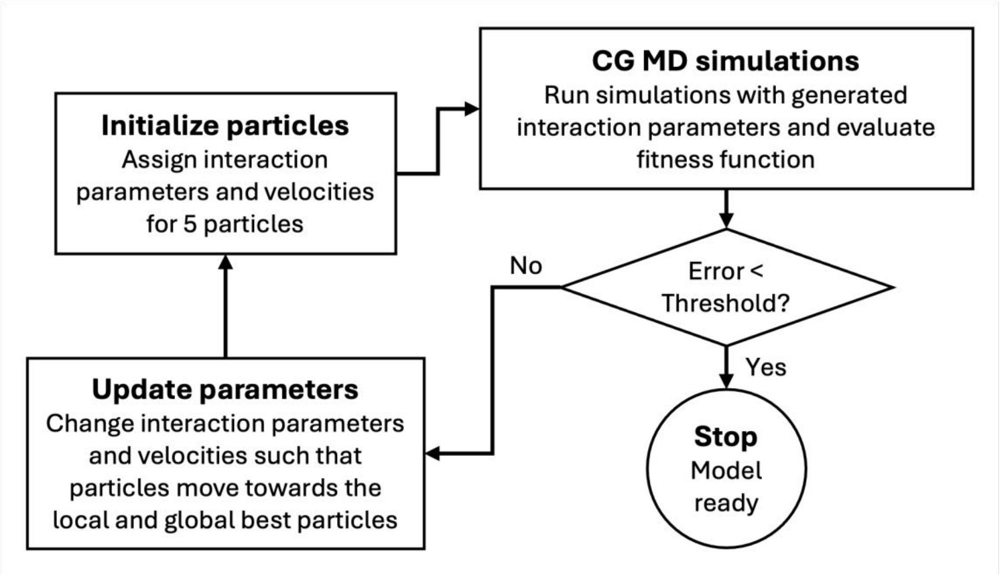

Figure S1: Schematic showing the Particle Swarm Optimization (PSO) algorithm.

Nonbonded parameter optimization was carried out by conducting coarse-grained (CG) molecular dynamics (MD) simulations guided by the PSO method, a powerful optimization algorithm inspired by the social behavior of bird flocking or fish schooling.[10] PSO operates by iteratively adjusting a population of candidate solutions, known as particles, based on their performance in the optimization problem, efficiently converging to optimal solutions.[11–13] Similar to our previous work,[11–19] here PSO was used to update the CG MD interaction parameters

through iterative lipid simulations while reducing the error between simulated and experimentally measured lipid properties. In the beginning of the PSO algorithm, randomly initialized particles (consisting of interaction parameters within predefined ranges) are generated, each with an arbitrary initial velocity. The velocity is adjusted by the algorithm, every epoch, to change the interaction parameters associated with each bird as the optimization proceeds. The new velocity is calculated based on the best-known local solution from that epoch (P_{best}) and the global best-known solution found across the optimization run (G_{best}). To prevent overshooting, i.e. to prevent a parameter set from deviating too much from its existing set of values, a maximum velocity limit (V_{max} = 0.02) is imposed. In each iteration, the fitness of each particle (set of parameters) is evaluated by comparing the calculated values with the experimental target values. If the particles get stuck in local minima for 20 consecutive iterations, all particles except the best one (least error) are re-scattered to improve their chances of finding minimum error parameter sets. Optimization was continued for 200 epochs, or till the obtained CG properties were close to the experimental targets without much chance of improvement.

Table S1: Optimized bonded interaction parameters used in this study.

**Bond**	**Force Constant** (kcal/mol/Å^2)	**Equilibrium bond length** (Å)
C2E-C3M	18.0	3.01
C2M-C3M	18.0	3.03
C2E-C2M	22.0	2.50
C3M -C3M	14.0	3.55
C3E-C3M	15.0	3.55
C3M-D3M	10.0	3.33
C3M-MTF	20.0	3.20
C3M-COH	15.0	2.80
CCO-COH	10.0	2.82
CCO-MTF	10.0	3.56
CCO-PO2	25.0	2.66
CHO-CCO	45.0	2.7
**Angle**	**Force Constant** (kcal/mol/radian^2)	**Equilibrium angle** (°)
C3M-C2M-C2E	3.30	147.0
C3M-C3M-C2E	3.30	147.0

C3M-C3M-C2M	3.30	147.0
C3M-C3M-D3M	3.30	145.0
C3M-C3M-C3M	3.30	147.0
C3M-C3M-C3E	3.30	147.0
C3M-D3M-C3M	2.80	130.9
C3M-C3M-MTF	3.00	140.0
C3M-C3M-COH	3.00	137.0
C3M-COH-CCO	4.00	140.0
C3M-MTF-CCO	3.00	135.0
COH-CCO-MTF	6.00	68.5
PO2-CCO-COH	3.00	130.0
PO2-CCO-MTF	3.00	136.0
CCO-PO2-CCO	7.00	90.0
PO2-CCO-CHO	8.00	125.5

Table S2: Optimized nonbonded interaction parameters used in this study. Initial ranges for parameter optimization are provided in the parenthesis.

**Self-Interaction**	**ε** (kcal/mol)	**σ** (Å)
CHO	0.7327 *(0.5, 1.0)*	6.2042 *(6.05, 6.35)*
PO2	0.7217 *(0.5, 0.9)*	5.8259 *(5.60, 5.90)*
D3M	0.5478 *(0.5, 0.6)*	4.7519 *(4.65, 4.85)*
COH	0.9767 *(0.7, 1.1)*	5.5218 *(5.35, 5.70)*
MTF	0.7620 *(0.7, 1.0)*	4.3740 *(4.25, 4.65)*
**Cross-Interaction**	**ε** (kcal/mol)	**σ** (Å)
CHO-W1	0.7573 *(0.75, 1.05)*	4.2710 *(4.0, 5.5)*
PO2-W1	0.8700 *(0.75, 1.01)*	3.8041 *(3.5, 5.0)*
COH-W1	0.9475 *(0.89, 1.12)*	3.9765 *(3.5, 4.5)*
MTF-W1	0.9967 *(0.89, 1.07)*	4.1604 *(3.5, 4.5)*

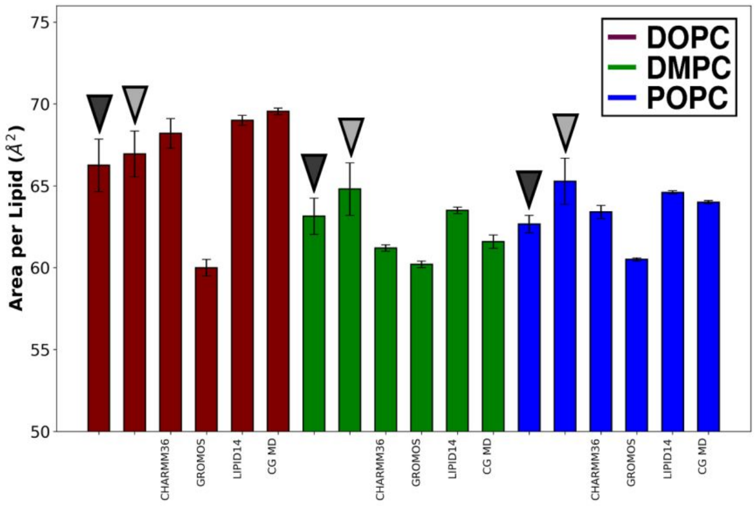

Figure S2: Comparison of A_L for DOPC, DMPC, and POPC bilayers. Bars marked with dark/light grey pointers indicate our experimental and CG MD simulation values, respectively. Remaining bars represent values obtained using other atomistic and CG FFs reported in literature. Refer to **Table 2** in the **main manuscript**.

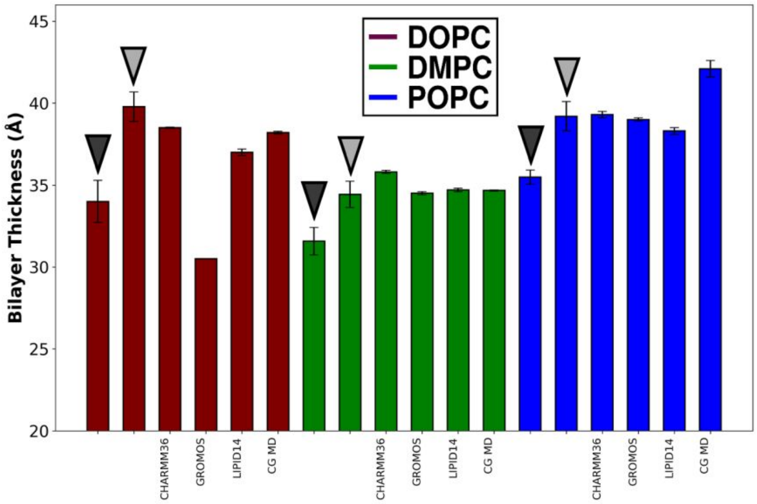

Figure S3: Comparison of D_{PP} for DOPC, DMPC, and POPC bilayers. Bars marked with dark/light grey pointers indicate our experimental and CG MD simulation values, respectively. Remaining bars represent values obtained using other atomistic and CG FFs reported in literature. Refer to **Table 2** in the **main manuscript**.

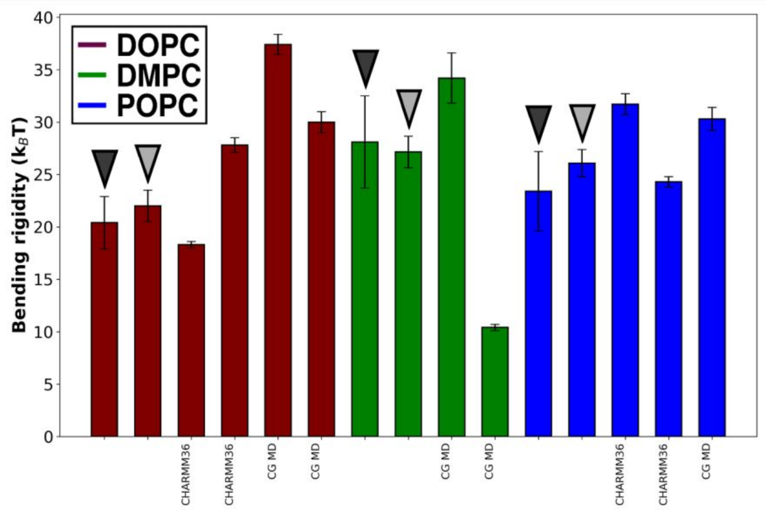

Figure S4: Comparison of κ for DOPC, DMPC, and POPC bilayers. Bars marked with dark/light grey pointers indicate our experimental and CG MD simulation values, respectively. Remaining bars represent values obtained using other atomistic and CG FFs reported in literature. Refer to **Table 2** in the **main manuscript**.

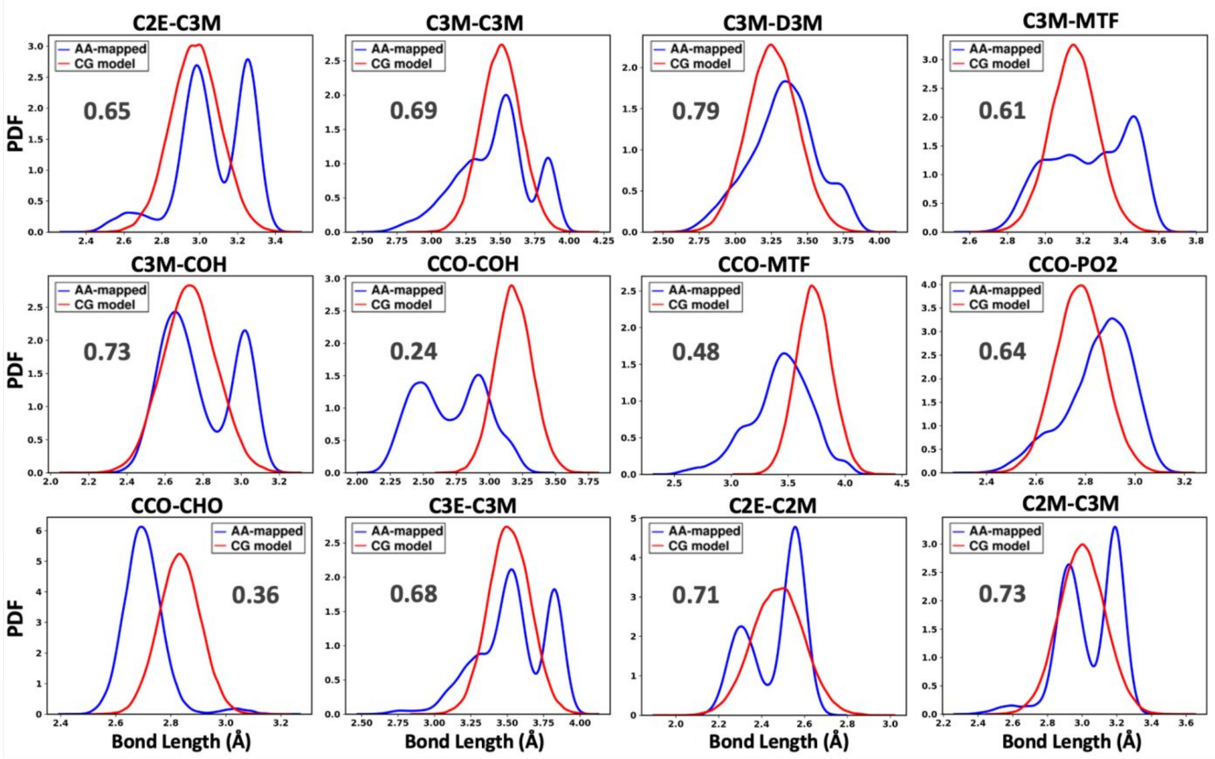

Figure S5: Probability density functions (PDFs) for bond lengths for the CG lipid model (red) developed in this work compared against mapped PDFs from atomistic simulations (blue). Refer to **Fig. 1** in the **main manuscript** for mapping scheme and bead definitions. Overlap coefficient values that provide quantitative measure of similarity between the PDFs, presented in each plot.

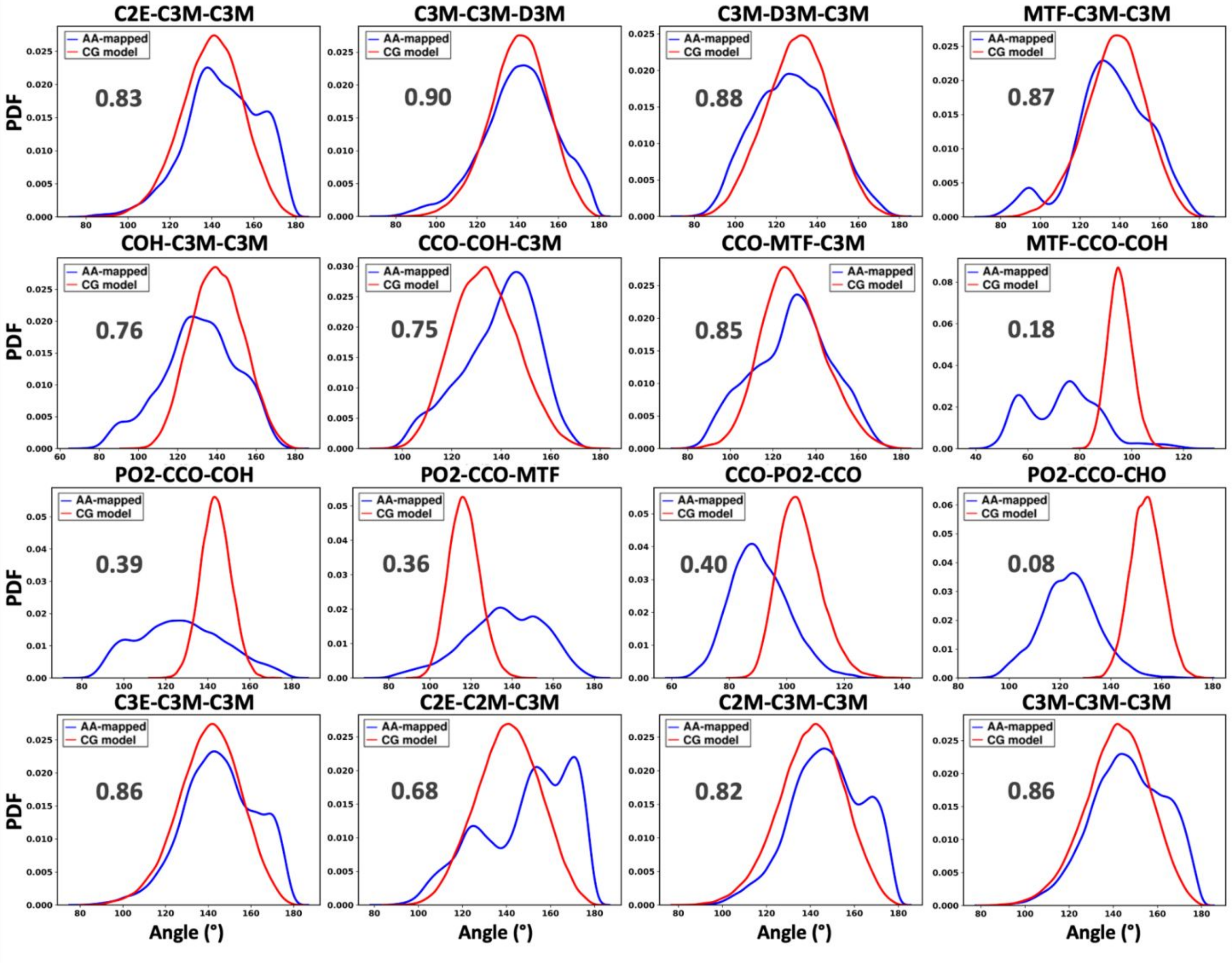

Figure S6: Probability density functions (PDFs) for angles for the CG lipid model (red) developed in this work compared against mapped PDFs from atomistic simulations (blue). Refer to **Fig. 1** in the **main manuscript** for mapping scheme and bead definitions. Overlap coefficient values that provide quantitative measure of similarity between the PDFs, presented in each plot.

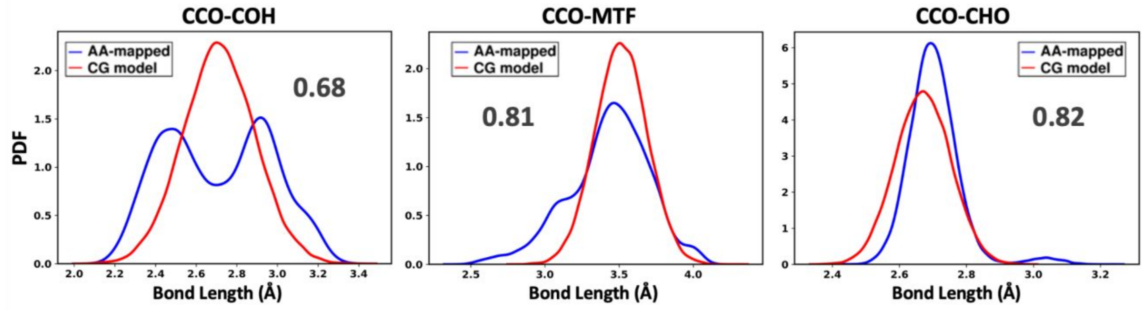

Figure S7: Representative bond length PDFs for the CG lipid model (red) with "exclude 1-3" compared with the mapped PDFs from atomistic simulations (blue). Compare these to PDFs from **Fig. S5** which were obtained using the "exclude 1-2" criteria. Overlap coefficient values that provide quantitative measure of similarity between the PDFs, presented in each plot.

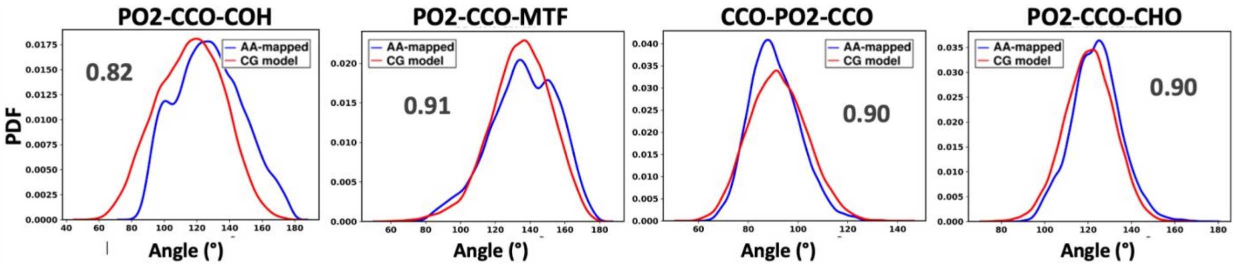

Figure S8: Representative angle PDFs for CG lipid model (red) with "exclude 1-3" compared with the mapped PDFs from atomistic simulations (blue). Compare these to PDFs from **Fig. S6** which were obtained using the "exclude 1-2" criteria. Overlap coefficient values that provide quantitative measure of similarity between the PDFs, presented in each plot.

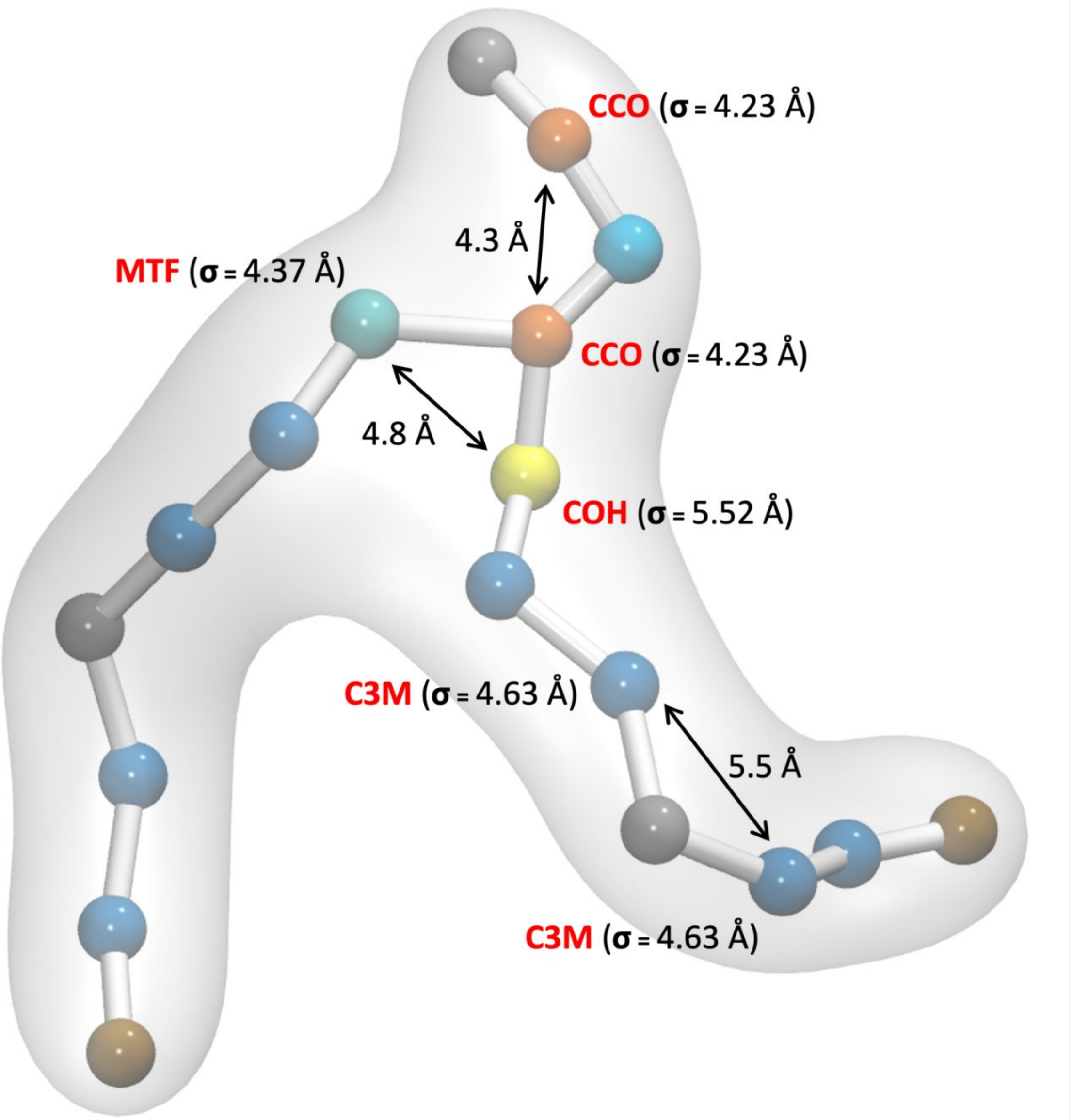

Figure S9: Representative structure of CG DOPC showing the competing effects between bonded and nonbonded interactions resulting in shifted bond and angle distributions.

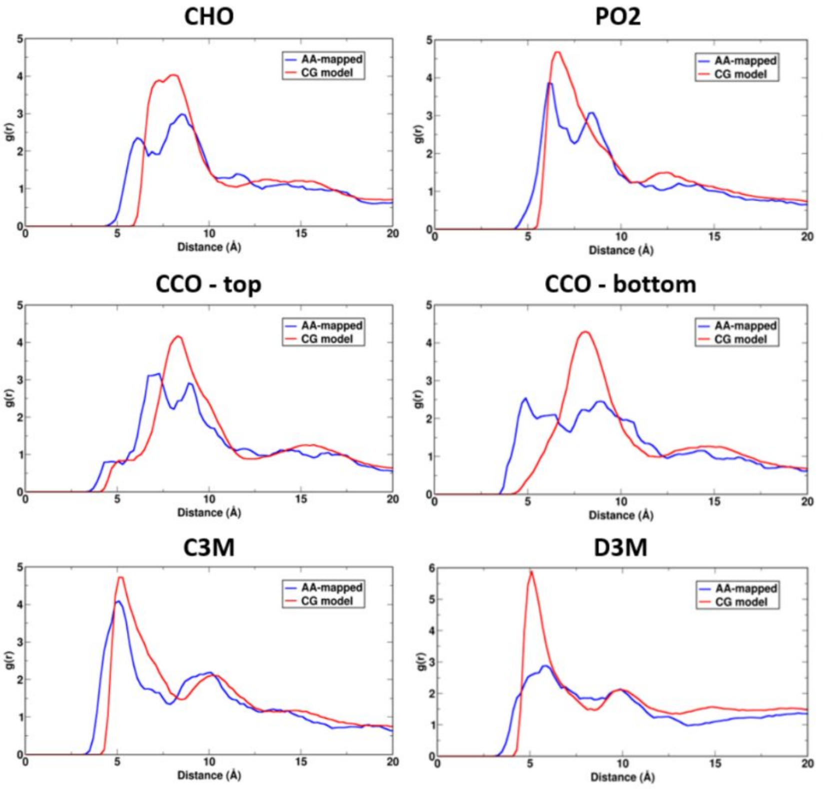

Figure S10: Radial Distribution Functions (RDFs) for CG lipid beads (red) compared with the RDFs from atomistic simulations (blue).

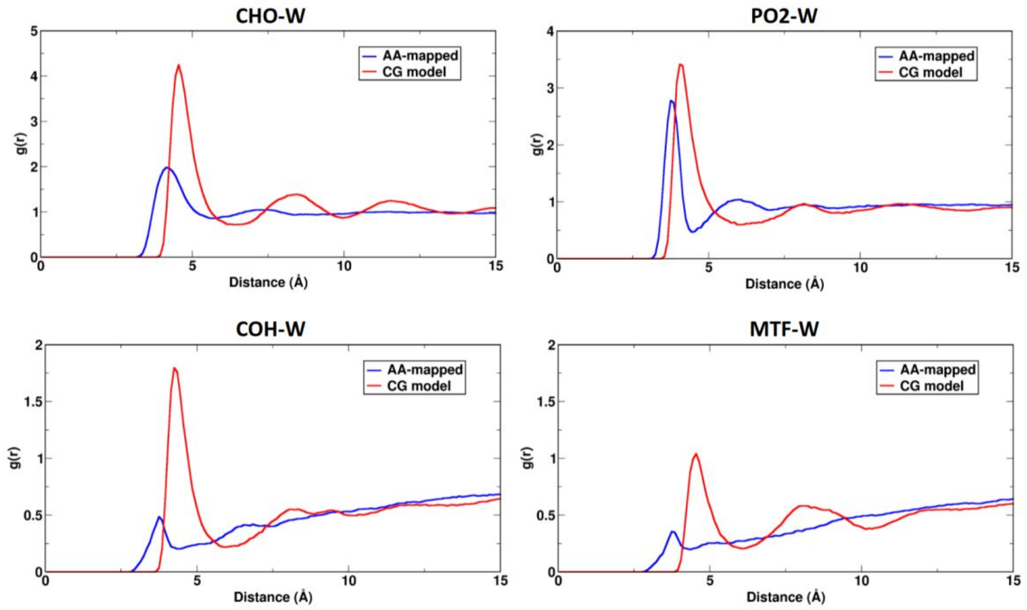

Figure S11: Radial Distribution Functions (RDFs) between newly parameterized CG lipid beads and water (red) compared with the mapped RDFs from atomistic simulations (blue).

Table S3: Calculated A_L values for DOPC, POPC, and DMPC with multiple system sizes and at different stages in the simulation run. All values are reported in $Å^2$ units.

Runtime	**System Size** (No.of lipids)								
	**DOPC**			**POPC**			**DMPC**		
	**128**	**288**	**576**	**128**	**288**	**576**	**128**	**288**	**576**
40-50 ns	66.8 ± 0.7	65.8 ± 0.6	65.5 ± 0.5	65.3 ± 0.7	65.5 ± 0.5	65.1 ± 0.3	65.2 ± 0.9	64.8 ± 0.6	64.9 ± 0.4
90-100 ns	66.8 ± 0.5	65.6 ± 0.5	65.7 ± 0.4	65.4 ± 0.9	65.4 ± 0.5	65.3 ± 0.5	64.5 ± 0.8	64.7 ± 0.7	64.5 ± 0.4
140-150 ns	67.2 ± 0.7	65.6 ± 0.6	65.7 ± 0.5	65.1 ± 0.5	65.6 ± 0.4	65.3 ± 0.4	64.7 ± 0.8	64.9 ± 0.7	64.6 ± 0.5
190-200 ns	67.0 ± 0.8	65.6 ± 0.6	65.9 ± 0.5	65.3 ± 0.6	65.6 ± 0.6	65.2 ± 0.3	64.7 ± 0.6	64.5 ± 0.5	64.8 ± 0.4
**Mean**	66.9 ± 1.4	65.6 ± 1.2	65.7 ± 1.0	65.3 ± 1.4	65.5 ± 1.0	65.2 ± 0.8	64.8 ± 1.6	64.7 ± 1.3	64.7 ± 0.9

Table S4: Calculated D_{PP} values for DOPC, POPC, and DMPC with multiple system sizes and at different stages in the simulation run. All values are reported in Å units.

Runtime	**System Size** (No.of lipids)								
	**DOPC**			**POPC**			**DMPC**		
	**128**	**288**	**576**	**128**	**288**	**576**	**128**	**288**	**576**
40-50 ns	39.9 ± 0.5	39.9 ± 0.3	40.1 ± 0.3	39.2 ± 0.5	39.1 ± 0.3	39.4 ± 0.2	34.3 ± 0.5	34.5 ± 0.3	34.5 ± 0.2
90-100 ns	39.8 ± 0.5	40.1 ± 0.3	40.0 ± 0.2	39.1 ± 0.4	39.2 ± 0.3	39.3 ± 0.3	34.5 ± 0.4	34.4 ± 0.3	34.6 ± 0.2
140-150 ns	39.7 ± 0.4	40.1 ± 0.3	40.0 ± 0.3	39.3 ± 0.5	39.2 ± 0.3	39.4 ± 0.2	34.5 ± 0.4	34.5 ± 0.4	34.6 ± 0.2
190-200 ns	39.7 ± 0.4	40.0 ± 0.3	39.9 ± 0.3	39.2 ± 0.3	39.2 ± 0.2	39.4 ± 0.2	34.4 ± 0.3	34.6 ± 0.2	34.5 ± 0.2
**Mean**	39.8 ± 0.9	40.0 ± 0.6	40.0 ± 0.6	39.2 ± 0.9	39.2 ± 0.6	39.4 ± 0.5	34.4 ± 0.8	34.5 ± 0.6	34.6 ± 0.4

Table S5: Calculated κ values for DOPC, POPC, and DMPC with multiple system sizes and at different stages in the simulation run. All values are reported in k_BT units.

Runtime	**System Size** (No.of lipids)								
	**DOPC**			**POPC**			**DMPC**		
	**128**	**288**	**576**	**128**	**288**	**576**	**128**	**288**	**576**
40-50 ns	23.6 ± 1.1	23.3 ± 0.2	21.4 ± 0.1	24.8 ± 0.6	26.0 ± 0.4	25.3 ± 0.3	25.7 ± 0.7	24.0 ± 0.2	25.1 ± 0.3
90-100 ns	22.0 ± 0.7	25.1 ± 0.6	24.3 ± 0.3	28.1 ± 0.6	24.6 ± 0.4	26.3 ± 0.4	29.0 ± 0.8	26.0 ± 0.6	26.4 ± 0.5
140-150 ns	22.1 ± 0.5	24.2 ± 0.4	22.2 ± 0.4	26.1 ± 0.8	27.2 ± 0.3	24.9 ± 0.4	26.6 ± 0.7	26.5 ± 0.4	26.9 ± 0.5
190-200 ns	20.3 ± 0.5	23.8 ± 0.5	21.8 ± 0.4	25.3 ± 0.5	25.9 ± 0.5	25.2 ± 0.3	27.3 ± 0.7	28.5 ± 0.5	27.6 ± 0.4
**Mean**	22.0 ± 1.5	24.1 ± 0.9	22.4 ± 0.7	26.1 ± 1.3	25.9 ± 0.8	25.4 ± 0.7	27.2 ± 1.5	26.3 ± 0.9	26.5 ± 0.9

Table S6: Calculated A_L values for DOPC, POPC, and DMPC with different CG MD time steps and at different stages in the simulation run. All values are reported in $Å^2$ units.

Run time	**Simulation time-step (fs)**														
	**DOPC**					**POPC**					**DMPC**				
	**5**	**10**	**15**	**20**	**25**	**5**	**10**	**15**	**20**	**25**	**5**	**10**	**15**	**20**	**25**
40-50 ns	66.1 ± 0.7	66.8 ± 0.7	66.4 ± 0.7	67.1 ± 0.9	65.7 ± 0.5	65.8 ± 0.6	65.3 ± 0.7	65.3 ± 0.8	65.0 ± 0.7	64.5 ± 0.6	64.8 ± 0.8	65.2 ± 0.9	65.4 ± 0.9	65.1 ± 1.0	65.2 ± 1.1
90-100 ns	65.4 ± 0.6	66.8 ± 0.5	65.5 ± 0.9	66.1 ± 0.8	66.4 ± 0.8	65.5 ± 0.6	65.4 ± 0.9	64.8 ± 0.6	64.5 ± 0.8	65.7 ± 0.8	65.4 ± 0.9	64.5 ± 0.8	64.5 ± 0.9	64.1 ± 0.9	62.9 ± 0.7
140-150 ns	65.1 ± 0.6	67.2 ± 0.7	65.0 ± 0.8	65.6 ± 0.8	67.6 ± 0.7	64.7 ± 0.5	65.1 ± 0.5	65.2 ± 0.6	66.0 ± 0.9	64.8 ± 0.6	63.9 ± 0.8	64.7 ± 0.8	64.6 ± 0.7	64.8 ± 0.9	63.2 ± 0.7
190-200 ns	65.2 ± 0.6	67.0 ± 0.8	65.0 ± 0.7	66.8 ± 0.8	65.9 ± 0.9	65.1 ± 0.6	65.3 ± 0.6	64.2 ± 0.6	66.3 ± 0.9	65.4 ± 0.5	64.2 ± 0.9	64.7 ± 0.6	63.7 ± 0.8	65.1 ± 0.9	65.9 ± 0.9
**Mean**	65.5 ± 1.3	67.0 ± 1.4	65.5 ± 1.6	66.4 ± 1.7	66.4 ± 1.5	65.3 ± 1.2	65.3 ± 1.4	64.9 ± 1.3	65.4 ± 1.7	65.1 ± 1.3	64.6 ± 1.7	64.8 ± 1.6	64.6 ± 1.7	64.8 ± 1.9	64.3 ± 1.7

Table S7: Calculated D_{PP} values for DOPC, POPC, and DMPC with different CG MD time steps and at different stages in the simulation run. All values are reported in Å units.

Run time	Simulation time-step (fs)														
	DOPC					POPC					DMPC				
	5	10	15	20	25	5	10	15	20	25	5	10	15	20	25
40-50 ns	40.0 ± 0.5	39.9 ± 0.5	39.5 ± 0.4	39.4 ± 0.4	39.6 ± 0.5	39.5 ± 0.4	39.2 ± 0.5	38.7 ± 0.4	37.7 ± 0.4	38.6 ± 0.3	34.3 ± 0.4	34.3 ± 0.5	34.2 ± 0.4	34.0 ± 0.4	34.0 ± 0.4
90-100 ns	39.7 ± 0.4	39.8 ± 0.5	39.8 ± 0.5	39.4 ± 0.4	39.7 ± 0.5	39.3 ± 0.5	39.1 ± 0.4	39.2 ± 0.4	39.0 ± 0.5	38.9 ± 0.4	34.2 ± 0.4	34.5 ± 0.4	33.8 ± 0.5	33.6 ± 0.4	33.9 ± 0.4
140-150 ns	39.8 ± 0.4	39.7 ± 0.4	39.2 ± 0.5	39.5 ± 0.4	39.6 ± 0.4	38.9 ± 0.4	39.3 ± 0.5	39.1 ± 0.4	39.0 ± 0.4	39.0 ± 0.5	33.9 ± 0.3	34.5 ± 0.4	34.3 ± 0.4	34.1 ± 0.4	34.0 ± 0.4
190-200 ns	39.5 ± 0.4	39.7 ± 0.4	39.4 ± 0.4	39.3 ± 0.4	39.5 ± 0.4	39.3 ± 0.3	39.2 ± 0.3	39.1 ± 0.4	38.7 ± 0.5	39.0 ± 0.4	34.3 ± 0.4	34.4 ± 0.3	34.5 ± 0.5	34.3 ± 0.4	34.2 ± 0.3
**Mean**	39.8 ± 0.9	39.8 ± 0.9	39.5 ± 0.9	39.4 ± 0.8	39.6 ± 0.9	39.3 ± 0.8	39.2 ± 0.9	39.0 ± 0.8	38.6 ± 0.9	38.9 ± 0.8	34.2 ± 0.8	34.4 ± 0.8	34.2 ± 0.9	34.0 ± 0.8	34.0 ± 0.8

Table S8: Calculated κ values for DOPC, POPC, and DMPC with different CG MD time steps and at different stages in the simulation run. All values are reported in k_BT units.

Run time	Simulation time-step (fs)														
	DOPC					POPC					DMPC				
	5	10	15	20	25	5	10	15	20	25	5	10	15	20	25
40-50 ns	24.4 ± 0.5	23.6 ± 1.1	21.9 ± 0.8	23.5 ± 0.6	23.6 ± 0.9	23.5 ± 0.6	24.8 ± 0.6	27.1 ± 0.6	25.9 ± 0.5	27.2 ± 0.3	24.5 ± 0.4	25.7 ± 0.7	24.7 ± 0.4	25.3 ± 0.6	26.5 ± 0.9
90-100 ns	23.1 ± 0.8	22.0 ± 0.7	23.4 ± 0.8	25.2 ± 0.9	21.9 ± 0.7	25.9 ± 0.7	28.1 ± 0.6	26.4 ± 0.6	27.8 ± 0.6	25.4 ± 0.6	25.1 ± 0.5	29.0 ± 0.8	23.9 ± 0.6	25.4 ± 0.7	25.8 ± 0.7
140-150 ns	22.0 ± 0.7	22.1 ± 0.5	24.4 ± 0.5	22.3 ± 0.7	25.5 ± 0.7	26.0 ± 0.3	26.1 ± 0.8	26.6 ± 0.5	24.3 ± 0.8	25.3 ± 0.7	29.4 ± 0.9	26.6 ± 0.7	26.5 ± 0.7	26.8 ± 0.8	28.6 ± 0.8
190-200 ns	23.2 ± 0.9	20.3 ± 0.5	26.7 ± 0.4	22.0 ± 0.5	22.2 ± 0.7	25.3 ± 0.7	25.3 ± 0.5	29.0 ± 0.8	28.6 ± 0.6	25.3 ± 0.6	28.7 ± 0.7	27.3 ± 0.7	27.6 ± 0.7	25.9 ± 0.6	28.6 ± 0.7
**Mean**	23.2 ± 1.5	22.0 ± 1.5	24.1 ± 1.3	23.3 ± 1.4	23.3 ± 1.5	25.2 ± 1.2	26.1 ± 1.3	27.3 ± 1.3	26.7 ± 1.3	25.8 ± 1.1	26.9 ± 1.3	27.2 ± 1.5	25.7 ± 1.2	25.9 ± 1.4	27.4 ± 1.6

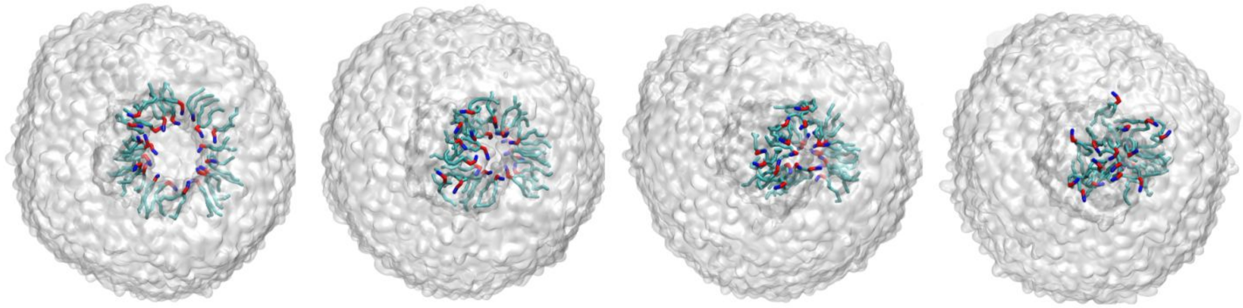

Figure S12: Snapshots exhibiting the sealing of the vesicle pore in the DOPC simulation. The entire vesicle is represented by a white semi-transparent surface whereas key lipid molecules near the pore are represented as licorice.

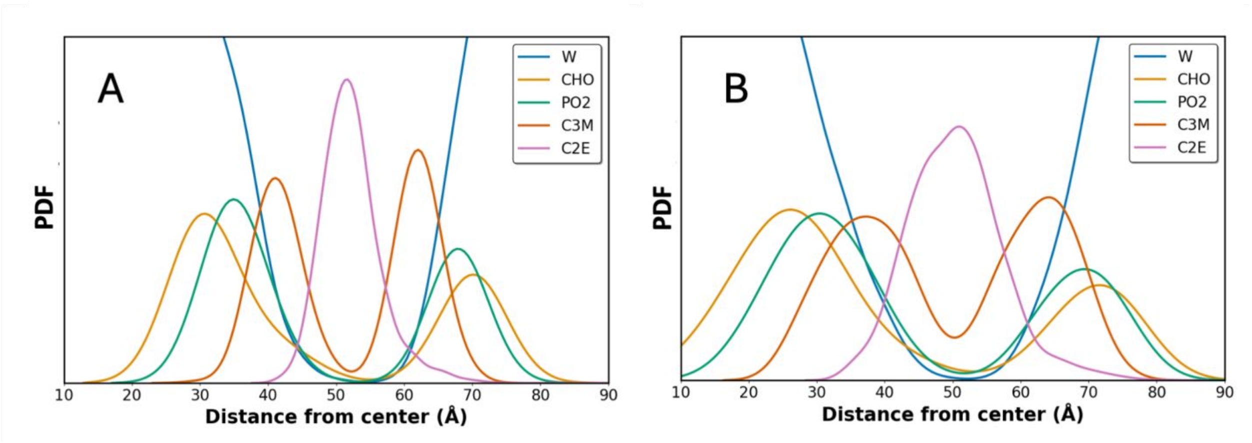

Figure S13: Normalized probability density functions of W, CHO, PO2, first C3M, and C2E beads, with respect to the center of the assembled vesicle for **A)** DMPC, and **B)** POPC vesicles.

Table S9: Calculated A_L and hydrophobic thickness ($2D_C$) values for DLPC, DMPC, DPPC, and DSPC membranes simulated at temperatures ranging from 303 to 353 K using the developed CG model without reparameterization. Values in the parenthesis were obtained using AA MD simulations and reported by Zhuang *et al.*[20] Simulations below the transition temperature were not performed by the cited study.

**Transition temp (K)**	~271		~297		~315		~328	
	**DLPC (12:0)**		**DMPC (14:0)**		**DPPC (16:0)**		**DSPC (18:0)**	
**Temp (K)**	$\mathbf{A_L}$ (Å^2)	$\mathbf{2D_C}$ (Å)	$\mathbf{A_L}$ (Å^2)	$\mathbf{2D_C}$ (Å)	$\mathbf{A_L}$ (Å^2)	$\mathbf{2D_C}$ (Å)	$\mathbf{A_L}$ (Å^2)	$\mathbf{2D_C}$ (Å)

**303**	62.0 (63.1)	19.2 (21.2)	62.9 (60.2)	22.9 (25.3)	63.2	25.6	64.2	29.3
**313**	64.8 (63.3)	19.0 (21.1)	64.8 (62.1)	22.1 (25.1)	64.7	25.3	64.9	29.2
**323**	65.2 (64.3)	18.8 (21.0)	65.3 (63.1)	21.9 (24.9)	65.4 (61.8)	24.3 (28.9)	67.2	28.5
**333**	66.5 (66.0)	18.6 (20.7)	67.5 (65.0)	21.8 (24.6)	69.7 (63.4)	24.6 (28.5)	67.8 (61.8)	27.7 (33.1)
**343**	68.5 (66.8)	18.2 (20.8)	67.0 (66.0)	18.5 (24.5)	68.9 (65.1)	24.4 (28.2)	68.2 (63.4)	27.0 (32.4)
**353**	70.2 (68.2)	18.0 (20.6)	68.3 (67.3)	21.0 (24.2)	71.9 (65.7)	23.6 (28.2)	68.5 (66.1)	27.4 (31.4)

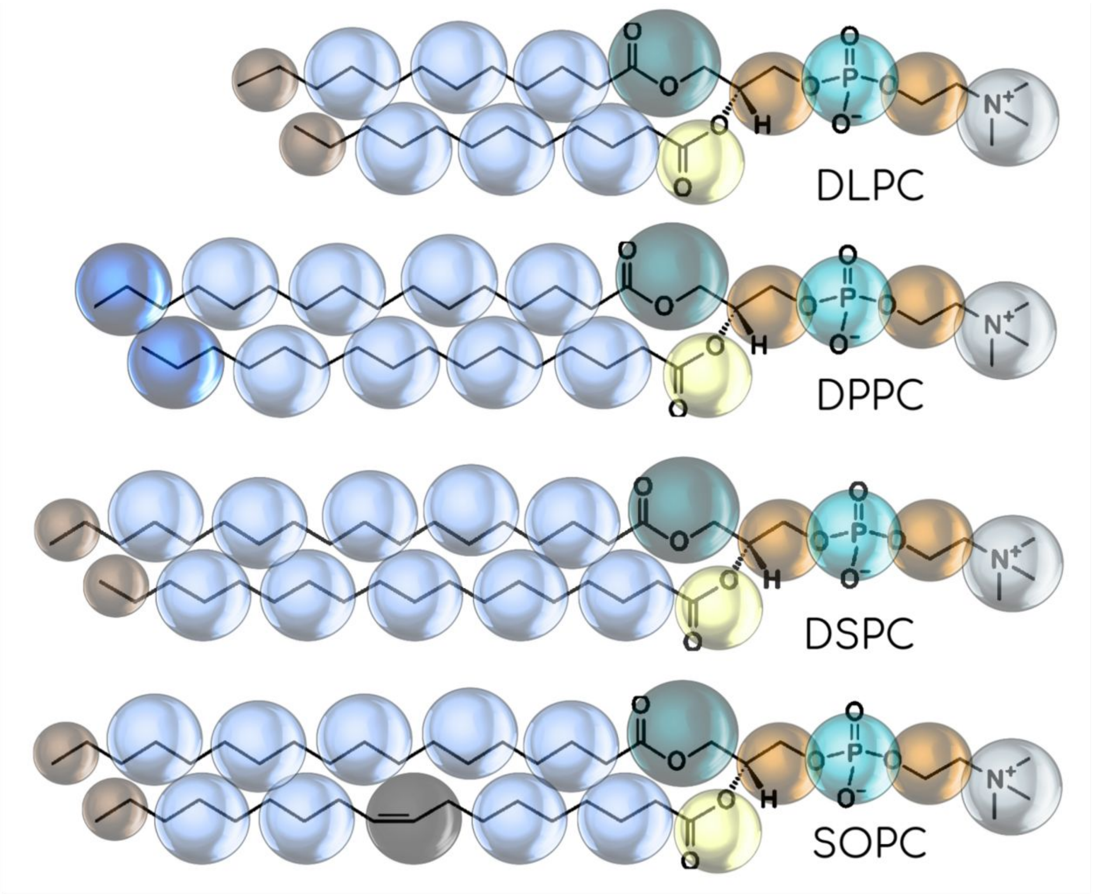

Figure S14: Mapping schemes for the lipid molecules (DLPC, DPPC, DSPC, and SOPC) using the CG beads optimized in this work. Beads with the same color correspond to the same bead type. Refer to **Fig. 1** in the **Main manuscript** for bead types.

*** Topology file for CG LIPIDS**

```
MASS   -1      CHO    59.11
MASS   -1      CCO    44.07
MASS   -1      PO2    62.97
MASS   -1      COH    30.04
MASS   -1      MTF    58.05
MASS   -1      C3M    42.09
MASS   -1      D3M    42.09
MASS   -1      C2E    29.07
MASS   -1      C2M    28.07
MASS   -1      C3E    43.09

RESI DOPC        0.00 !
GROUP
ATOM  CHO   CHO    0.00
ATOM  H1    CCO    0.00
ATOM  PO2   PO2    0.00
ATOM  H2    CCO    0.00
ATOM  SA    COH    0.00
ATOM  SB    MTF    0.00
ATOM  C1A   C3M    0.00
ATOM  C2A   C3M    0.00
ATOM  C3A   D3M    0.00
ATOM  C4A   C3M    0.00
ATOM  C5A   C3M    0.00
ATOM  C6A   C2E    0.00
ATOM  C1B   C3M    0.00
ATOM  C2B   C3M    0.00
ATOM  C3B   D3M    0.00
ATOM  C4B   C3M    0.00
ATOM  C5B   C3M    0.00
ATOM  C6B   C2E    0.00
```

```
BOND CHO H1  H1 PO2  PO2 H2  H2 SA
BOND H2 SB  SA C1A  C1A C2A  C2A C3A
BOND C3A C4A  C4A C5A  C5A C6A  SB C1B
BOND C1B C2B  C2B C3B  C3B C4B  C4B C5B
BOND C5B C6B

RESI DMPC         0.00 !
GROUP
ATOM  CHO   CHO    0.00
ATOM  H1    CCO    0.00
ATOM  PO2   PO2    0.00
ATOM  H2    CCO    0.00
ATOM  SA    COH    0.00
ATOM  SB    MTF    0.00
ATOM  C1A   C3M    0.00
ATOM  C2A   C3M    0.00
ATOM  C3A   C3M    0.00
ATOM  C4A   C2M    0.00
ATOM  C5A   C2E    0.00
ATOM  C1B   C3M    0.00
ATOM  C2B   C3M    0.00
ATOM  C3B   C3M    0.00
ATOM  C4B   C2M    0.00
ATOM  C5B   C2E    0.00

BOND CHO H1  H1 PO2  PO2 H2  H2 SA
BOND H2 SB  SA C1A  C1A C2A  C2A C3A
BOND C3A C4A  C4A C5A  SB C1B
BOND C1B C2B  C2B C3B  C3B C4B  C4B C5B

RESI POPC         0.00 !
GROUP
ATOM  CHO   CHO    0.00
ATOM  H1    CCO    0.00
```

```
ATOM  PO2   PO2   0.00
ATOM  H2    CCO   0.00
ATOM  SA    COH   0.00
ATOM  SB    MTF   0.00
ATOM  C1A   C3M   0.00
ATOM  C2A   C3M   0.00
ATOM  C3A   D3M   0.00
ATOM  C4A   C3M   0.00
ATOM  C5A   C3M   0.00
ATOM  C6A   C2E   0.00
ATOM  C1B   C3M   0.00
ATOM  C2B   C3M   0.00
ATOM  C3B   C3M   0.00
ATOM  C4B   C3M   0.00
ATOM  C5B   C3M   0.00

BOND CHO H1  H1 PO2  PO2 H2  H2 SA
BOND H2 SB  SA C1A  C1A C2A  C2A C3A
BOND C3A C4A  C4A C5A  C5A C6A  SB C1B
BOND C1B C2B  C2B C3B  C3B C4B  C4B C5B

END
```

*** Force Field parameters for CG LIPIDS**

BONDS

C2E	C3M	18.000	3.010
C2M	C3M	18.000	3.030
C2E	C2M	22.000	2.500
C3M	C3M	14.000	3.550
C3E	C3M	15.000	3.550
C3M	D3M	10.000	3.330
D3M	D3M	10.000	3.300
C3M	MTF	20.000	3.200
C3M	COH	15.000	2.800
D3M	COH	15.000	2.800
CCO	COH	10.000	2.820
CCO	MTF	10.000	3.560
CCO	PO2	25.000	2.660
CHO	CCO	45.000	2.700

ANGLES

C3M	C2M	C2E	3.3000	147.000
C3M	C3M	C2E	3.3000	147.000
D3M	C3M	C2E	3.3000	147.000
C3M	C3M	C2M	3.3000	147.000
C3M	C3M	D3M	3.3000	145.000
C3M	C3M	C3M	3.3000	147.000
C3M	C3M	C3E	3.3000	147.000
C3M	D3M	C3M	2.8000	130.902
C3M	D3M	D3M	2.5000	130.902
D3M	D3M	D3M	2.5000	130.902
C3M	C3M	MTF	3.0000	140.000
C3M	C3M	COH	3.0000	137.000
D3M	D3M	COH	2.5000	130.902
C3M	COH	CCO	4.0000	140.000
D3M	COH	CCO	4.0000	140.000
C3M	MTF	CCO	3.0000	135.000

```
COH CCO MTF      6.0000       68.5000
PO2 CCO COH      3.0000       130.000
PO2 CCO MTF      3.0000       136.000
CCO PO2 CCO      7.0000       90.0000
PO2 CCO CHO      8.0000       125.500

DIHEDRALS
X  X  X  X     0.0     0.0     0.0

NONBONDED
W1     0.0     -1.142500     2.1170
CHO    0.0     -0.732739     3.4821
PO2    0.0     -0.721725     3.2698
C3M    0.0     -0.554500     2.6009
D3M    0.0     -0.547772     2.6670
C3E    0.0     -0.592700     2.6009
COH    0.0     -0.976735     3.0991
MTF    0.0     -0.761963     2.4549
C2E    0.0     -0.371000     2.4343
C2M    0.0     -0.342000     2.4343
CCO    0.0     -0.799900     2.3741

NBFIX
C3M    W1      -0.543400     4.5000
D3M    W1      -0.543400     4.5000
C2E    W1      -0.513000     4.2363
C2M    W1      -0.440000     4.2363
C3E    W1      -0.620200     4.5000
COH    W1      -0.947528     4.4636
MTF    W1      -0.996701     4.6700
CCO    W1      -1.008149     4.4911
CHO    W1      -0.757266     4.7942
PO2    W1      -0.870000     4.2701

END
```

References

(1) Kumarage, T.; Nguyen, J.; Ashkar, R. Neutron Spin Echo Spectroscopy as a Unique Probe for Lipid Membrane Dynamics and Membrane-Protein Interactions. *J. Vis. Exp.* **2021**, No. 171. https://doi.org/10.3791/62396.

(2) Scott, H. L.; Skinkle, A.; Kelley, E. G.; Waxham, M. N.; Levental, I.; Heberle, F. A. On the Mechanism of Bilayer Separation by Extrusion, or Why Your LUVs Are Not Really Unilamellar. *Biophys. J.* **2019**, *117* (8), 1381–1386.

(3) *SasView*. http://www.sasview.org/ (accessed 2024-07-25).

(4) Chakraborty, S.; Doktorova, M.; Molugu, T. R.; Heberle, F. A.; Scott, H. L.; Dzikovski, B.; Nagao, M.; Stingaciu, L.-R.; Standaert, R. F.; Barrera, F. N.; Katsaras, J.; Khelashvili, G.; Brown, M. F.; Ashkar, R. How Cholesterol Stiffens Unsaturated Lipid Membranes. *Proceedings of the National Academy of Sciences* **2020**, *117* (36), 21896–21905.

(5) Azuah, R. T.; Kneller, L. R.; Qiu, Y.; Tregenna-Piggott, P. L. W.; Brown, C. M.; Copley, J. R. D.; Dimeo, R. M. DAVE: A Comprehensive Software Suite for the Reduction, Visualization, and Analysis of Low Energy Neutron Spectroscopic Data. *J. Res. Natl. Inst. Stand. Technol.* **2009**, *114* (6), 341–358.

(6) Zilman, A. G.; Granek, R. Undulations and Dynamic Structure Factor of Membranes. *Phys. Rev. Lett.* **1996**, *77* (23), 4788–4791.

(7) Watson, M. C.; Brown, F. L. H. Interpreting Membrane Scattering Experiments at the Mesoscale: The Contribution of Dissipation within the Bilayer. *Biophys. J.* **2010**, *98* (6), L9–L11.

(8) Nagao, M.; Kelley, E. G.; Ashkar, R.; Bradbury, R.; Butler, P. D. Probing Elastic and Viscous Properties of Phospholipid Bilayers Using Neutron Spin Echo Spectroscopy. *J. Phys. Chem. Lett.* **2017**, *8* (19), 4679–4684.

(9) Hoffmann, I.; Michel, R.; Sharp, M.; Holderer, O.; Appavou, M.-S.; Polzer, F.; Farago, B.; Gradzielski, M. Softening of Phospholipid Membranes by the Adhesion of Silica Nanoparticles--as Seen by Neutron Spin-Echo (NSE). *Nanoscale* **2014**, *6* (12), 6945–6952.

(10) Kennedy, J.; Eberhart, R. Particle Swarm Optimization. In *Proceedings of ICNN'95 - International Conference on Neural Networks*; 1995; Vol. 4, pp 1942–1948 vol.4.

(11) Bejagam, K. K.; Singh, S.; An, Y.; Berry, C.; Deshmukh, S. A. PSO-Assisted Development of New Transferable Coarse-Grained Water Models. *J. Phys. Chem. B* **2018**, *122* (6), 1958–1971.

(12) An, Y.; Bejagam, K. K.; Deshmukh, S. A. Development of New Transferable Coarse-Grained Models of Hydrocarbons. *J. Phys. Chem. B* **2018**, *122* (28), 7143–7153.

(13) Bejagam, K. K.; An, Y.; Singh, S.; Deshmukh, S. A. Machine-Learning Enabled New Insights into the Coil-to-Globule Transition of Thermosensitive Polymers Using a Coarse-Grained Model. *J. Phys. Chem. Lett.* **2018**, *9* (22), 6480–6488.

(14) An, Y.; Bejagam, K. K.; Deshmukh, S. A. Development of Transferable Nonbonded Interactions between Coarse-Grained Hydrocarbon and Water Models. *J. Phys. Chem. B* **2019**, *123* (4), 909–921.

(15) Conway, O.; An, Y.; Bejagam, K. K.; Deshmukh, S. A. Development of Transferable Coarse-Grained Models of Amino Acids. *Molecular Systems Design & Engineering* **2020**, *5* (3), 675–685.

(16) Mohammadi, E.; Joshi, S. Y.; Deshmukh, S. A. Development, Validation, and Applications of Nonbonded Interaction Parameters between Coarse-Grained Amino Acid and Water Models. *Biomacromolecules* **2023**, *24* (9), 4078–4092.

(17) An, Y.; Singh, S.; Bejagam, K. K.; Deshmukh, S. A. Development of an Accurate Coarse-Grained Model of Poly(acrylic Acid) in Explicit Solvents. *Macromolecules* **2019**, *52* (13), 4875–4887.

(18) Bejagam, K. K.; Singh, S.; An, Y.; Deshmukh, S. A. Machine-Learned Coarse-Grained

Models. *J. Phys. Chem. Lett.* **2018**, *9* (16), 4667–4672.
(19) Joshi, S. Y.; Deshmukh, S. A. A Review of Advancements in Coarse-Grained Molecular Dynamics Simulations. *Mol. Simul.* **2021**, *47* (10-11), 786–803.
(20) Zhuang, X.; Dávila-Contreras, E. M.; Beaven, A. H.; Im, W.; Klauda, J. B. An Extensive Simulation Study of Lipid Bilayer Properties with Different Head Groups, Acyl Chain Lengths, and Chain Saturations. *Biochim. Biophys. Acta* **2016**, *1858* (12), 3093–3104.
